# Sbg1 Is a Novel Regulator for the Localization of the β-Glucan Synthase Bgs1 in Fission Yeast

**DOI:** 10.1371/journal.pone.0167043

**Published:** 2016-11-29

**Authors:** Reshma Davidson, Josef A. Pontasch, Jian-Qiu Wu

**Affiliations:** 1 Department of Molecular Genetics, The Ohio State University, Columbus, Ohio, United States of America; 2 Department of Biological Chemistry and Pharmacology, The Ohio State University, Columbus, Ohio, United States of America; Tokyo Daigaku, JAPAN

## Abstract

Glucan synthases synthesize glucans, complex polysaccharides that are the major components in fungal cell walls and division septa. Studying regulation of glucan synthases is important as they are essential for fungal cell survival and thus popular targets for anti-fungal drugs. Linear 1,3-β-glucan is the main component of primary septum and is synthesized by the conserved β-glucan synthase Bgs1 in fission yeast cytokinesis. It is known that Rho1 GTPase regulates Bgs1 catalytic activity and the F-BAR protein Cdc15 plays a role in Bgs1 delivery to the plasma membrane. Here we characterize a novel protein Sbg1 that is present in a complex with Bgs1 and regulates its protein levels and localization. Sbg1 is essential for contractile-ring constriction and septum formation during cytokinesis. Sbg1 and Bgs1 physically interact and are interdependent for localization to the plasma membrane. Bgs1 is less stable and/or mis-targeted to vacuoles in *sbg1* mutants. Moreover, Sbg1 plays an earlier and more important role in Bgs1 trafficking and localization than Cdc15. Together, our data reveal a new mode of regulation for the essential β-glucan synthase Bgs1 by the novel protein Sbg1.

## Introduction

Cell walls protect a cell from environmental stresses, withstand high internal turgor pressure, and maintain cell shape. Cytokinesis in fungi requires the coordination between a constricting actomyosin contractile ring and a growing external cell-wall structure, the septum. The ring is important for the positioning, shaping, and orderly growth of the septum [1–6]. In many fungal species including the fission yeast *Schizosacchromyces pombe*, the cell wall and the septum mainly consist of polysaccharide glucans [7,8]. The septum is a trilaminar structure with a primary septum sandwiched by two secondary septa. During cell separation, the primary septum is digested by glucanases and the secondary septa becomes the new cell wall of daughter cells [9].

The main component of the primary septum in fission yeast is the linear β-1,3-glucan synthesized by glucan synthase Bgs1/Cps1 [10,11]. The secondary septum consists of 1,6 branched β-1,3-glucans synthesized by Bgs4, α-1,3-glucans synthesized by Ags1, and β-1,6-glucans [12]. β-1,6-glucans are relatively short polymers of glucose (~350 glucose units) linked by β-1,6-glycosidic linkages [8]. The synthases for β-1,6-glucans have not been definitely identified but Kre6 and Skn1 are important for the elongation of β-1,6-glucan chains in budding yeast cell wall [13–15]. The glucan synthases Bgs1, Bgs4, and Ags1 are essential transmembrane proteins delivered to the plasma membrane via secretory pathway [11,16–20].

The regulation of glucan synthases is of keen interest as they are essential for cytokinesis and cell-wall formation, making them ideal targets for anti-fungal drugs with minimal side effects on plant and animal cells including humans [21,22]. Several proteins have been documented for regulating glucan synthases in yeast and filamentous fungi. Rho1 GTPase activates β-1,3-glucan synthases as a regulatory subunit [23–26]. The α-glucan synthase Ags1 is regulated by Rho1 and Rho2 GTPases via the protein kinase C [27,28]. The F-BAR protein Cdc15 scaffolds several proteins involved in cytokinesis at the division site and maintains the contractile ring stability during anaphase. In *cdc15* mutants, Bgs1 trafficking from Golgi to the division site is impeded [1]. Rho-GAP Rga7 plays a role in β-1,3-glucan synthase Bgs4 delivery from Golgi to the cleavage site during cytokinesis [29,30]. The clathrin coat proteins (COPs) were also involved in glucan synthase regulation. In yeast, a mutation in the *SOO1* gene, encoding the α-COP protein Soo1, affects β-glucan synthase activity and displays an osmo-sensitive phenotype. Soo1 is likely involved in the transport of proteins involved in β-glucan synthesis from ER to Golgi [31]. In *A*. *nidulans*, the α-COP protein Sod^VI^Cp plays a role in cell wall synthesis and protein secretion by affecting β-glucan synthase activity [32]. Moreover, *S*. *pombe* clathrin light chain *clc1* deletion mutant is defective in Bgs1 localization [33]. However, none of the multi-functional proteins are essential for Bgs1 or Bgs4 localization.

Here we investigated the roles of the essential protein Sbg1 in cytokinesis and revealed a novel mode of regulation for the β-glucan synthase Bgs1. Sbg1 is a binding partner of Bgs1 and is essential for Bgs1 localization and stability. We also find that Sbg1 has a more important role than the F-BAR protein Cdc15 in Bgs1 trafficking and localization.

## Results

### *sbg1* mutant has abnormally stable contractile rings and fails cytokinesis

We first developed an interest in the uncharacterized protein Sbg1 (SPBP22H7.03) after a genome-wide deletion screen in *S*. *pombe* categorized it as an essential gene with putative function in cytokinesis [34]. It was reported that *sbg1*Δ led to long branched cells and Sbg1 localized to the division site [34,35]. Sbg1 is a small protein (20 kDa) with one recognizable SKN1 domain, which contains a transmembrane helix and is similar to a small portion of β-glucan synthesis associated proteins of SKN1 and KRE6 proteins in yeast (S1A Fig). SKN1 and KRE6 play redundant roles in synthesis and incorporation of β-1,6-glucan in the fungal cell wall [14,15]. The Sbg1 SKN1 homology domain is conserved in SKN1 and KRE6 proteins of many pathogenic fungal species such as *Candida* and *Aspergillus* (S1B Fig). All of the homologs have putative roles in β-1,6-glucan synthesis (S1C Fig). However, their roles in regulating other glucan synthases have not been reported.

**Fig 1:**
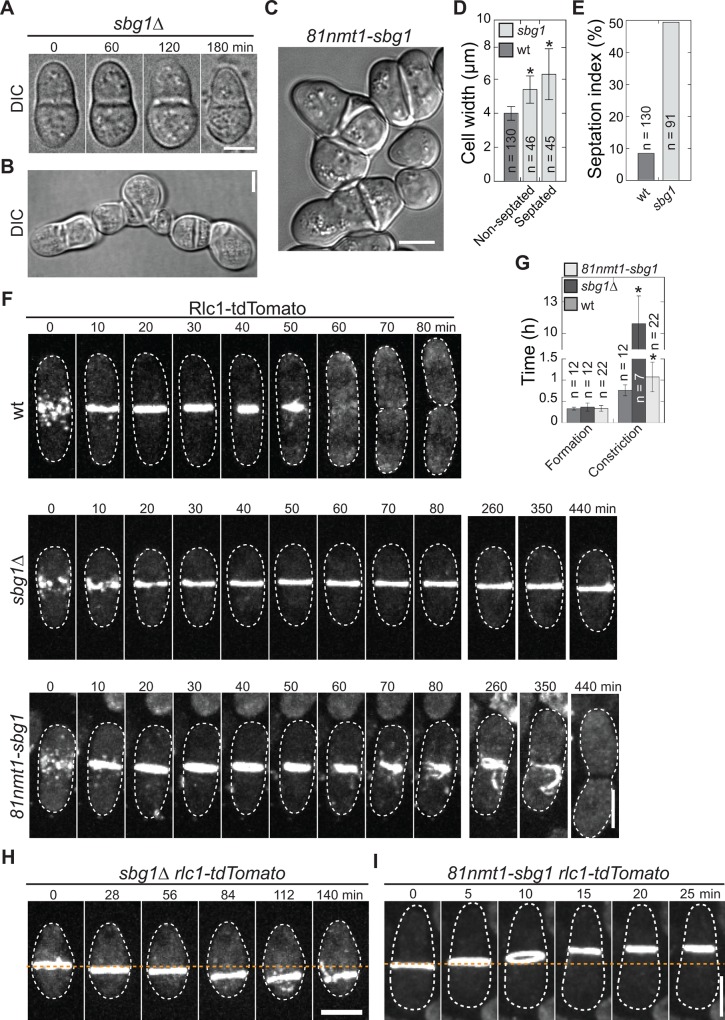
*sbg1* mutants are defective in contractile-ring constriction and septation. Germinated spores in (A, F, and H) were imaged 12–24 h after tetrad dissection onto YE5S plates. (A) Time course of a *sbg1*Δ cell lysed at 180 min. (B) A *sbg1*Δ cell germinated on YE5S + sorbitol and imaged after 3 d of growth. (C-E) Micrograph (C) and quantifications (D and E) of *sbg1* depletion phenotype of strain *81nmt1-mECitrine-sbg1* grown in YE5S + thiamine for 60 h. (D) Cell width at the widest part of the cell perpendicular to cell long axis. In this and other figures, asterisks indicate p < 0.01 in Student’s *t*-test and error bars are ± 1 SD. (E) Percentage of cells with ≥1 septa. (F and G) Contractile-ring formation and constriction in wt and *sbg1* mutants. *81nmt1-sbg1 rlc1-tdTomato* and control cells were grown in YE5S + thiamine liquid cultures for 36 h before imaging. Time 0 marks node appearance. (G) Timing of ring formation (node appearance to a compact ring) and constriction (a compact ring to ring constricted to a dot with highest Rlc1 pixel intensity, which includes the ring-maturation stage) in wt and *sbg1* mutants. (H and I) Ring anchoring is defective in *sbg1* mutants. Ring sliding in (H) *sbg1*Δ and (I) *81nmt1-sbg1* cells. (I) Cells were grown as in (F). Scale bars (for this and other figures except EM): 5 μm.

To determine the role of Sbg1 in cytokinesis and cell-wall formation, we deleted one copy of *sbg1* in a diploid strain carrying homozygous tandem dimer Tomato (tdTomato)-tagged myosin regulatory light chain Rlc1 as a contractile-ring marker. The spores separated with a 2:2 ratio with two dead as single cells confirming that Sbg1 is an essential gene. *sbg1*Δ spores germinated normally but arrested with septa, and some lysed while attempting cytokinesis (Fig 1A, S2A Fig and S1 Movie). *sbg1*Δ cells that survived longer gradually lost polarity and became swollen (S2A and S2B Fig). Sorbitol partially rescued *sbg1*Δ cells and *sbg1*Δ cells germinated on media with sorbitol survived for up to 3 days. However, cytokinesis still failed and cell morphology was defective in these cells, which were elongated, multi-septated, and swollen (Fig 1B and S2 Movie). The cell-lysis phenotype along with the mild rescue by sorbitol suggests that *sbg1*Δ cells have defective cell wall and/or septum.

Despite much effort, we could not obtain conditional *sbg1* mutants with strong phenotype using marker reconstitution mutagenesis [36], thus we depleted Sbg1 using thiamine repressible *nmt1* promotors [37,38]. Under repressing condition, Sbg1 levels were greatly reduced in *81nmt1-mECitrine* (monomeric enhanced Citrine)*-sbg1* cells and in *41nmt1-mECitrine-sbg1* cells (S2C Fig, arrows). The cells were rounder, multi-septated, or branched (Fig 1C), which were similar to *sbg1*Δ cells. The *81nmt1-sbg1* cells were wider than wild-type (wt) cells (Fig 1D), but they had no significant difference in cell length (S2D Fig). The mutant cells also had a higher septation index (~50% compared with ~9% in wt; Fig 1E) and ~8% cells were multiseptated, indicating a delay in cytokinesis.

We compared contractile-ring defects in *sbg1*Δ (using tetrad fluorescence microscopy) and *81nmt1-sbg1* cells using Rlc1-tdTomato as a marker (Fig 1F and 1G and S2B Fig). Ring formation and morphology appeared normal in both *sbg1* mutants. However, ring maturation and constriction were severely delayed. In wt cells ring maturation and constriction took 45 ± 8 min, whereas they took 663 ± 173 min in *sbg1*Δ cells. In 30% *sbg1*Δ cells (n = 13 cells) the ring did not constrict during the 13 h movies (Fig 1F middle, 1G, S2B Fig and S3 Movie). In *81nmt1-sbg1* cells under repressing conditions for 36 h, ring maturation and constriction were delayed and took 65 ± 21 min (Fig 1F and 1G). The ring was also not properly anchored in some *sbg1* mutant cells and slid along the cell long axis (Fig 1H and 1I). Thus, *sbg1* mutant phenotype suggests that Sbg1 likely plays a role in polarized growth, septum formation, ring constriction, and ring anchoring.

### Sbg1 plays a role in primary septum formation

**Fig 2:**
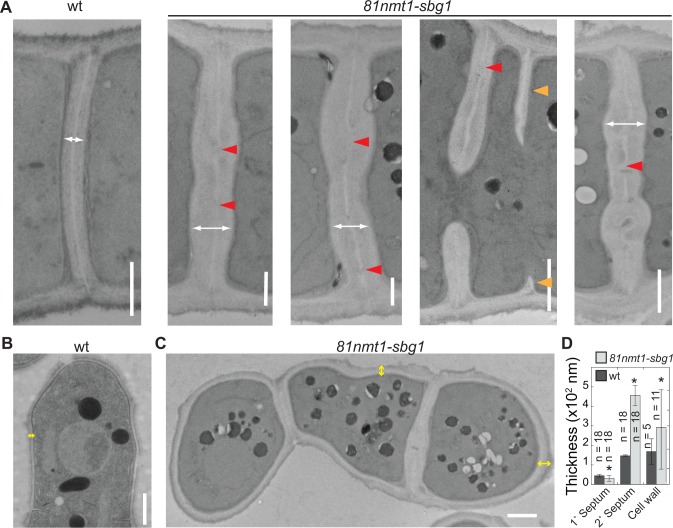
Sbg1 plays a role in primary septum formation. EM micrographs (A-C) and quantification (D) showing septum and cell-wall defects in *81nmt1-sbg1* cells. Double headed arrows indicate septa (white) or cell wall (yellow) thickness. Red arrowheads mark gaps in the primary septum. Orange arrowheads mark asymmetric septation. Scale bars: 1 μm.

The cell morphology, lysis, and contractile-ring defects of *sbg1* mutants resemble those in β-glucan synthase *bgs1* mutants. Defects in septum formation in *bgs1* mutants result in delayed contractile-ring constriction [11,39]. We examined cell wall and septum defects in *sbg1* mutant cells by Calcofluor staining, which stains the primary septa and growing cell tips [39]. Unlike wt cells, *81nmt1-mECitrine-sbg1* cells showing prominent septal structures in differential interference contrast (DIC) images were not stained well by Calcofluor (S2E Fig), suggesting a primary-septum defect similar to *bgs1* mutants [39]. Indeed, EM revealed that the primary septa in *81nmt1-sbg1* cells were discontinuous (Fig 2A, red arrowheads), uneven, and thinner (45 ± 7 nm in wt vs. 31 ± 15 nm in the mutant; Fig 2D). In addition, the septum grew asymmetrically in the mutant cells (Fig 2A, orange arrowheads). Thus, the contractile ring defects and the cytokinetic delay in *sbg1* mutants are likely due to failure in forming a fully functional primary septum. The secondary septa as well as the cell wall outside the division site in *81nmt1-sbg1* cells were ~3x and 2x as thick as those in wt (456 ± 52 nm vs. 147 ± 5 nm; Fig 2A–2D and S2F Fig). Taken together, the septum and cell-wall defects in *sbg1* depleted cells are similar to those in *bgs1* mutants [11,19,39], suggesting Sbg1 works in the same pathway as Bgs1 in primary-septum and cell-wall formation.

### Sbg1 co-localizes and interacts with the β-glucan synthase Bgs1

**Fig 3:**
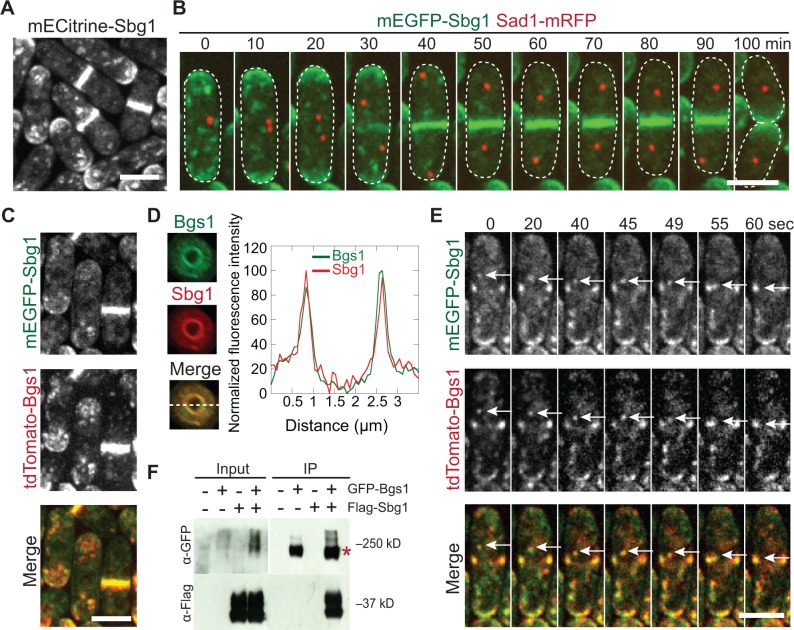
Sbg1 co-localizes and interacts with the β-glucan synthase Bgs1. (A) Sbg1 localization in cytoplasmic puncta, the division site, and cell tips. (B) Time course of arrival and ring formation of Sbg1 at the division site using Sad1 as a cell-cycle marker. (C-E) Micrograph (C), 3-D projection and line scans at the dashed line (D), and time course (E) showing co-localization of Sbg1 and Bgs1. (E) Single focal plane images of a representative cell showing Sbg1 and Bgs1 move together in cytoplasmic puncta (an example marked by white arrows). (F) Sbg1 co-IPs with Bgs1. IP using anti-GFP antibodies and cell lysates of strains expressing GFP-Bgs1, 3Flag-Sbg1, or GFP-Bgs1 3Flag-Sbg1 grown in YE5S for 48 h. The asterisk marks GFP-Bgs1. The smearing and multiple bands are likely caused by post-translational modifications.

To test whether Sbg1 and Bgs1 work together, we compared Sbg1 and Bgs1 localizations (Fig 3). Sbg1 localized to the division site, cell tips, and cytoplasmic puncta (Fig 3A and 3B). Using the spindle pole body (SPB) protein Sad1 as a cell-cycle marker, we determined that Sbg1 arrived at the division site during early anaphase B when SPBs were ~4 μm apart (Fig 3B and S3A Fig). Next we examined Sbg1 localization dependencies. Sbg1 partially depended on actin filaments for its localization. When treated with CK-666 (abolishing actin patches nucleated by the Arp2/3 complex) or Latrunculin-A (Lat-A; abolishing all actin filaments), Sbg1 intensity in cytoplasmic puncta decreased, more Sbg1 accumulated at cell tips, and it spread out to a wider region at the division site instead of a compact ring (S3B Fig). Sbg1 depended on the secretory pathway for efficient delivery to the cell tips and division site (S3C and S3D Fig). After Brefeldin-A (BFA) treatment, Sbg1 concentrations at the division site and cell tips were obviously reduced and cytoplasmic puncta mostly disappeared (S3C Fig and S4 Movie). Consistently, Sbg1 localization was affected by the mutation in vesicle-tether exocyst (S3D Fig; [40,41]). The temporal and spatial localization patterns and dependencies of Sbg1 localization are identical to those of Bgs1 [16,40].

Indeed, Sbg1 and Bgs1 colocalized in cytoplasmic puncta, cell tips, and the division site (Fig 3C and 3D). At the division site, they localized as a disc along the septum but were more concentrated near the leading edge of forming septum (Fig 3D). They traveled together in cytoplasmic puncta (Fig 3E, white arrows and S5 Movie). Thus, Sbg1 and Bgs1 might be delivered together to the division site by the secretory pathway. We also detected partial co-localization of Sbg1 with the other glucan synthases Bgs4 and Ags1 (S3E Fig). However, Sbg1 concentrated in the ring earlier than Bgs4 and Ags1 (S3E Fig, arrows). In addition, Bgs4 and Ags1 lagged behind Sbg1 during septum formation (S3F Fig). Together with the EM data, these results suggest that Sbg1 works together with Bgs1 in primary septum formation.

We used immunoprecipitation (IP) to test whether Sbg1 physically interacts with Bgs1. GFP-Bgs1 could pull down 3Flag-Sbg1 from *S*. *pombe* extracts of the strain expressing both proteins (Fig 3F). Thus, Sbg1 and Bgs1 are present in the same protein complex.

### Sbg1 and Bgs1 are interdependent for cellular localization

**Fig 4:**
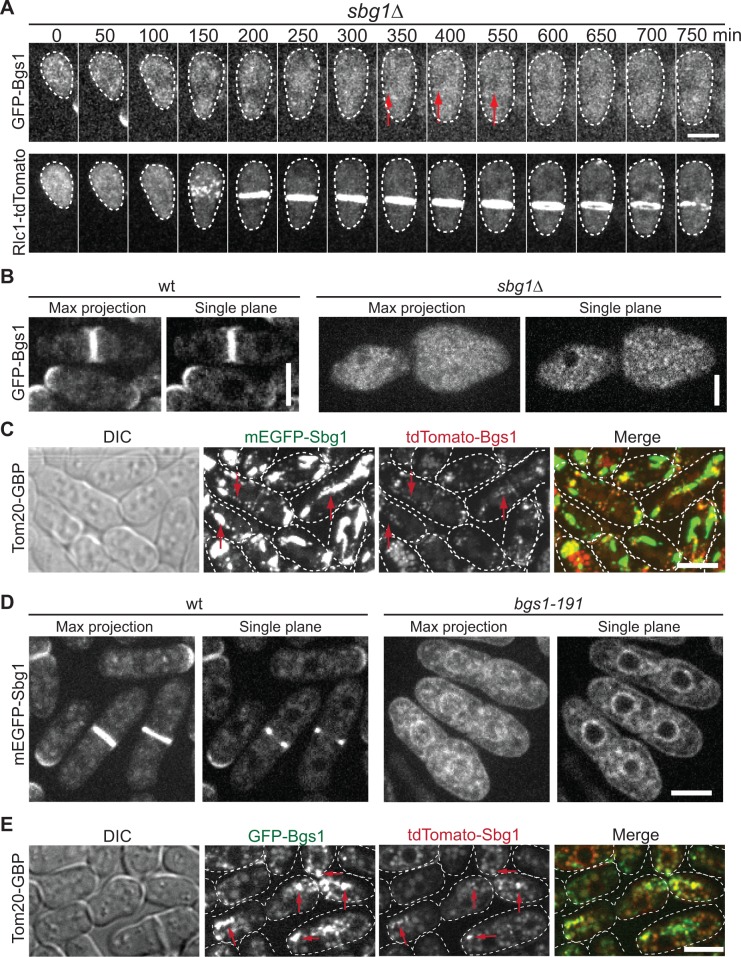
Sbg1 and Bgs1 are interdependent for localization. (A and B) Bgs1 localization depends on Sbg1. (A) Time course and (B) micrographs (left, maximum intensity projection; right, single focal plane) of *sbg1*Δ or *sbg1*^*+*^ cells expressing both tagged Bgs1 and Rlc1. (C) Mislocalized Sbg1 by Tom20-GBP recruits Bgs1 to mitochondria (examples marked by arrows). (D) Sbg1 depends on Bgs1 for division-site and cell-tip localization. Cells were grown at 36°C for 4 h. (E) Mislocalized Bgs1 by Tom20-GBP recruits Sbg1 to mitochondria (examples marked by arrows).

To determine the functional relationship between Sbg1 and Bgs1, we began by testing their localization dependence. Interestingly, Bgs1 localization depended on Sbg1 (Fig 4A and 4B; n = 53). The tip localization of Bgs1 was abolished in all *sbg1*Δ cells analyzed at different cell cycle stages and Bgs1 localization to the division site was greatly affected. ~70% of cells showed some residual Bgs1 signal in the Rlc1-labeled structures in time-lapse movies (Fig 4A, arrows, S4A Fig and S6 Movie). The residual signal was not bleedthrough from the red channel (S6C Fig). This suggests that some Bgs1 may still bind to the proteins in the contractile ring. However, no Bgs1 signal was seen at the membrane near growing or formed septal structures. The cytoplasm in *sbg1* mutants was very bright and GFP signal was seen in patch like structures, which were more obvious with higher imaging laser power (Fig 4B and S4C Fig). These data suggest that Sbg1 plays an important role in Bgs1 localization. In contrast, Sbg1 depletion had no obvious effect on Bgs4 and Ags1 localization (S4D and S4E Fig). To directly test the ability of Sbg1 to recruit Bgs1, we mislocalized Sbg1 to mitochondria using mitochondrial outer membrane protein Tom20 tagged with GFP binding protein (GBP; [42,43]). mEGFP-Sbg1 indeed recruited Bgs1 to the mitochondria (Fig 4C, arrows and S5A Fig). These data reveal that Sbg1 is a novel regulator of Bgs1 localization.

Surprisingly, Sbg1 also depended on Bgs1 for localization to the plasma membrane. In *bgs1* temperature-sensitive mutant at the restrictive temperature, Sbg1 appeared to be stuck in the nuclear membrane and endoplasmic reticulum (ER) and had no enrichment at the cell tips or the division site (Fig 4D). To test the ability of Bgs1 to recruit Sbg1, we mislocalized Bgs1 to the mitochondria using Tom20-GBP. Bgs1 could recruit Sbg1 to mitochondrial structures (Fig 4E, arrows and S5B Fig). Thus, Sbg1 and Bgs1 are interdependent for localization/targeting to the plasma membrane. Together, Sbg1 and Bgs1 may physically interact and co-transport to the plasma membrane for cell wall and septum synthesis.

### Sbg1 is important for Bgs1 stability

**Fig 5:**
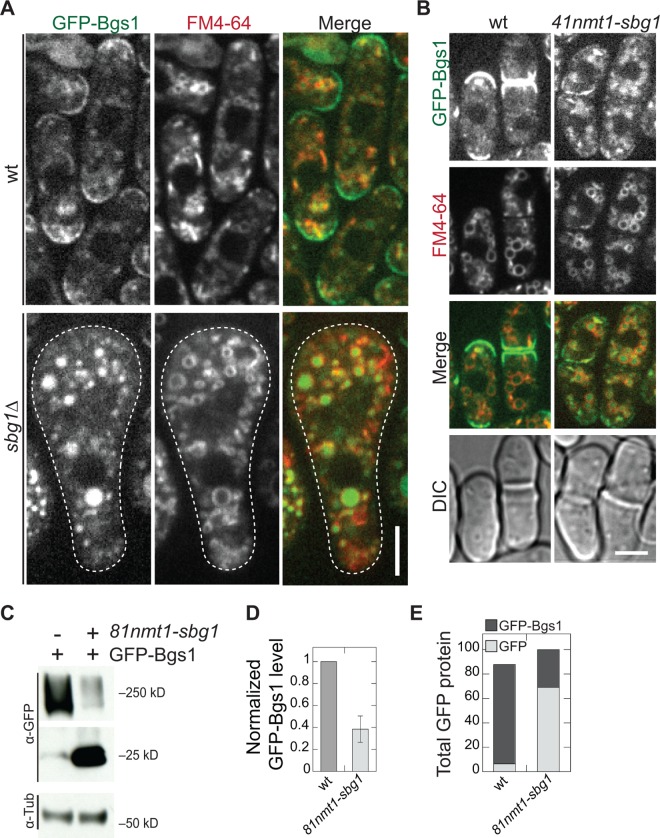
Sbg1 prevents the mistargeting of Bgs1 to vacuoles. (A and B) Bgs1 accumulates in vacuoles in *sbg1* mutants. FM4-64 staining of *sbg1*Δ and *sbg1*^*+*^ cells from spores germinated on YE5S plates for 24 h (A) or wt and *41nmt1-sbg1* cells grown in YE5S + thiamine for ~60 h (B). (C-E) Bgs1 degrades in *sbg1* mutant cells. (C) Western blotting and (D and E) quantifications (n = 6 from 2 independent experiments) of GFP-Bgs1 from cell lysates of *GFP-bgs1* and *81nmt1-sbg1 GFP-bgs1* cells grown in YE5S + thiamine for ~36 h.

To understand how Sbg1 regulates Bgs1, we further analyzed the Bgs1 localization in *sbg1* mutants with higher resolution. Unlike in *sbg1*^*+*^ cells, GFP-Bgs1 was absent from the plasma membrane at cell tips and the division site in *sbg1* mutants (Fig 5A and 5B). Bright GFP fluorescence signal was seen as patch like structures in the cytoplasm. These were identified as vacuoles, whose membrane was stained with FM4-64 dye, in *sbg1*Δ and *41nmt1*-*sbg1* depletion cells (Fig 5A and 5B). We confirmed the vacuolar localization of Bgs1 in *81nmt1-sbg1* depletion cells by staining vacuolar lumen with CMAC dye (S6A Fig; [44]). The GFP signals in vacuoles were not autofluorescence from sick *sbg1*Δ cells since they were absent in *sbg1*Δ cells without GFP-Bgs1 (S6B and S6C Fig).

The vacuolar fluorescence suggested that Bgs1 secretion to the plasma membrane is defective and/or Bgs1 is unstable without enough Sbg1. We tested whether Sbg1 plays a general role in secretory pathway or more specific to Bgs1 secretion. We detected no obvious defects in secretory pathway as the localizations of Sec8 (S6D Fig), a component of the vesicle tether exocyst complex; and Syb1 (S6E Fig), a v-SNARE on secretory vesicles were not affected in Sbg1 depletion cells. More importantly, the localizations of other secretory cargos Bgs4 and Ags1, two cell-wall glucan synthases, were not affected by Sbg1 depletion (S4D and S4E Fig). Together, these data suggest that Sbg1 plays a specific role in Bgs1 trafficking/stability and does not affect other secreted cargos.

Bgs1 accumulation in vacuoles also suggested Bgs1 destabilization from loss of Sbg1. We therefore tested Bgs1 levels in the *sbg1* mutant using Western blotting (Fig 5C–5E). The GFP-Bgs1 level in *81nmt1-sbg1* was reduced by ~60% compared to wt (Fig 5C and 5D). Interestingly, a prominent band at the size of free GFP was detected in the mutant but hardly in wt (Fig 5C). The total amount of GFP present in the mutant was similar to that in wt samples (Fig 5D and 5E). GFP is known to be very stable due to the presence of a stable fluorescence fold [45,46]. This suggests that in the *sbg1* mutants, Bgs1 but not the GFP tag was likely degraded. In contrast, the protein levels of Bgs4 and Ags1 were upregulated (S4F Fig). Together, these data suggest that Sbg1 plays an important role in maintaining Bgs1 protein levels and its trafficking.

### Sbg1 plays an earlier role than the F-BAR protein Cdc15 in Bgs1 localization

**Fig 6:**
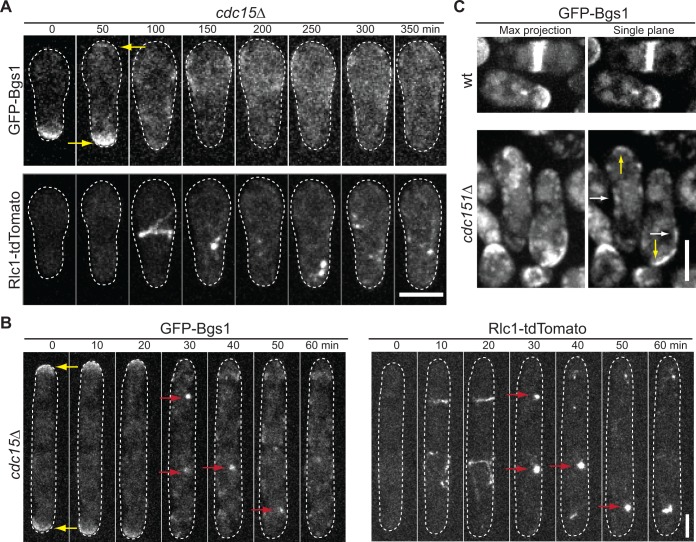
Roles of the F-BAR protein Cdc15 in Bgs1 localization. (A and B) Time course and (C) micrographs of Bgs1 localization in *cdc15*Δ or *cdc15*^*+*^ cells. (B) Time course showing Bgs1 and Rlc1 colocalization (arrows) in ~1/3 *cdc15*Δ cells.

The F-BAR protein Cdc15 is suggested to deliver Bgs1 from Golgi to the plasma membrane. In *cdc15* temperature sensitive mutant cells, localization of Bgs1 was affected [1]. Consistently, we found that Sbg1 localization was also affected in *cdc15* mutant cells (S6F Fig). We examined whether Sbg1 and Cdc15 play similar roles in Bgs1 trafficking to the plasma membrane. We observed GFP-Bgs1 localization in *cdc15*Δ cells using tetrad fluorescence microscopy. Unlike in *sbg1*Δ cells, Bgs1 localized to cell tips in *cdc15*Δ cells (Fig 6A–6C, yellow arrows; and S7 Movie). After the Rlc1 ring formed, Bgs1 delocalized from cell tips and spread on cell membrane unevenly (Fig 6C, single plane, white arrows). For 27 *cdc15*Δ cells observed during the ~11 h movies with 10 min interval, 52% cells had no Bgs1 concentration at the division site (Fig 6A); 33% of them had Bgs1 co-localizing with Rlc1 briefly after the ring collapse (Fig 6B, red arrows); the rest had neither Rlc1 nor Bgs1 localization at the division site. In both *sbg1*Δ and *cdc15*Δ cells, GFP-Bgs1 global level measured from GFP fluorescence intensity was similar to wt (S4B and S6G Figs). No excess accumulation of GFP signal in vacuoles was seen in *cdc15*Δ cells (S6H Fig). In *sbg1* mutants, Bgs1 cannot localize to cell tips or the plasma membrane (Fig 4A and 4B), which implies that Sbg1 plays a role in Bgs1 delivery to the plasma membrane prior to Cdc15 and Sbg1 has a role independent of Cdc15 and its interacting proteins in regulating Bgs1 localization. Together, Sbg1 plays a more important and earlier role than Cdc15 in Bgs1 delivery or localization.

## Discussion

In this study we find that Sbg1, a previously uncharacterized protein with a SKN1 domain from the Skn1/Kre6 family, plays a prominent role in regulating the β-glucan synthase Bgs1. Sbg1 binds to Bgs1 and regulates its localization to the cell tips and division site. We also find that Sbg1 is important for Bgs1 stability and trafficking.

### Roles of Sbg1 in regulating the glucan synthase Bgs1

The β-glucan synthase Bgs1 is essential for contractile-ring constriction and primary septum formation in fission yeast cytokinesis [16,39,40]. It localizes to the active growth sites: cell tips and the division site [16,40]. Our study reveals a novel mode of regulation on Bgs1 localization. In *sbg1*Δ cells, Bgs1 is unable to localize to the plasma membranes at cell tips or the division site. Instead, Bgs1 transiently co-localizes with misplaced contractile ring components and accumulates in vacuoles (Figs 4 and 5). This suggests a role for Sbg1 in Bgs1 trafficking.

The secretory pathway plays an important role in Bgs1 trafficking to the plasma membrane. In cells with mutation in Sip1, an accessory protein for the clathrin adaptor protein AP-1, Bgs1 fails to localize to cell tips and is partially accumulated in Golgi or endosomes near the division site and cell tips [41,47], which is different from Bgs1 localization seen in the *sbg1* mutants. The *sbg1* mutant phenotype is more similar to those of the clathrin light chain *clc1*Δ and the Rho GTPase *cdc42* mutants that compromise vesicle secretion and trafficking [33,48]. In these mutants, Bgs1, but not Bgs4, is mis-localized to the vacuoles. This indicates that Bgs1 and Bgs4 have different trafficking mechanisms, which is consistent with our data. However, in contrast to Clc1 and Cdc42 that are involved in several cellular processes [33], it seems that Sbg1 specifically affects only Bgs1 trafficking. Localizations of exocyst subunit Sec8, v-SNARE Syb1, and vesicle cargos Bgs4 and Ags1 are not obviously affected in *sbg1* mutant cells (S4D, S4E, S6D and S6E Figs). Thus, Sbg1 plays essential roles in Bgs1 trafficking and localization but does not affect the localization of secretory pathway components or other cargos tested in this study.

In *sbg1* mutants, we observed GFP signals in vacuoles. Western blotting showed majority GFP in Sbg1 depletion cells is free GFP and the level of GFP-Bgs1 dropped accordingly (Fig 5C–5E), suggesting that Bgs1 undergoes degradation. So we propose that Sbg1 may play a role in Bgs1 stability, acting as a chaperone/adaptor that allows successful secretion and function of Bgs1. This function is reminiscent of *S*. *cerevisiae* Keg1 that binds and stabilizes Kre6. In *keg1* mutant cells, Kre6 level but not its function can be restored by mutations in the vacuolar proteolysis pathway [13,49]. In *bgs1-191* mutant cells, no vacuolar localization or obvious loss in Sbg1 fluorescence was seen, Sbg1 was stuck in ER and/or nuclear envelope instead. This phenotype may be allele specific or Sbg1 only helps transport wild-type Bgs1 from ER to the plasma membrane. More studies are needed to distinguish these possibilities. Because Sbg1 remains colocalized with Bgs1 after Bgs1 arrives at its function sites, Sbg1 may also stabilize Bgs1 and its interactions with other proteins at the division site, as recently reported [Ref]. It has been proposed that β-glucan synthases mediated septum growth is mechanosensitive and coupled to contractile ring tension [3,4], so Sbg1 may also play a role in curvature-dependent septum growth.

It has been shown that the F-BAR protein Cdc15 is involved in Bgs1 delivery to the division site [1]. When Cdc15 is depleted, Bgs1 trafficking to the division site is slowed. Here we show that while Bgs1 localization to the division site is severely affected in *cdc15*Δ cells, Bgs1 can still localize to the membrane at cell tips and on the cell sides (Fig 6). Thus, the main function of Cdc15 may be to stabilize Bgs1 at the division site, to provide the cue for its recruitment through the contractile ring, or to play a role in the late stages of Bgs1 trafficking to the division site. Although it is likely that Bgs1 localization is regulated differently at the division site and cell tips, we propose that Sbg1 is more important for Bgs1 localization and function than Cdc15 for the following reasons. First, failure of Bgs1’s binding to the plasma membranes in *sbg1*Δ but not *cdc15*Δ cells indicates that Sbg1 functions earlier than Cdc15 in Bgs1 localization. Second, phenotype and primary-septum defects in *bgs1*Δ cells are similar to those in *sbg1*Δ but not in *cdc15*Δ cells. Third, Sbg1 and Bgs1 localization are interdependent and travel together to the division site. However, Cdc15 arrives at the division site much earlier [50] than Sbg1 (S3A Fig). Together, our data indicates that Sbg1 plays an earlier and more important role than Cdc15 in Bgs1 trafficking or membrane binding.

### Skn1/Kre6 family proteins function in glucan synthesis and may serve as potential anti-fungal drug targets

Skn1/Kre6 family proteins are well conserved in fungi including many pathogenic fungal species ([51]; S1 Fig). They were first isolated as mutants defective in the synthesis of β-1,6-glucans [52]. β-1,6-glucans are proposed to be important for the shape and stability of yeast cell wall and for anchoring several mannoproteins to the external cell wall [53]. In the absence of these proteins β-1,6-glucans have significantly reduced levels and are structurally defective [54]. The Skn1/Kre6 family proteins have not previously been shown to regulate β-1,3-glucan synthases. However, our *sbg1* mutants show defects consistent with those of *bgs1* mutants and Bgs1 fails to localize in *sbg1* mutants. Sbg1 only contains a transmembrane domain and part of the SKN1 domain of Skn1/Kre6 proteins. Thus, it is not surprising that Sbg1 has different function from Skn1/Kre6. C-terminal truncations of Sbg1 were inviable (our unpublished data), indicating that the SKN1 domain (and/or the embedded transmembrane domain) is essential for Sbg1 function. Sethi et al. [55] recently showed that a point mutation in the Skn1 domain of Sbg1 leads to decrease in cell wall β-1,3 glucan. Further studies are needed to determine the Sbg1 regions/domains involved in binding with Bgs1.

Orthologues of glucan synthases are absent in animal and plant cells, and thus have been robustly targeted for anti-fungal drug development [21,56]. Echinocandins and several derivatives target β-1,3-glucan synthesis [21,56]. However, recently a mutation hotspot was discovered in yeast β-1,3-glucan synthase Fks1 that confers resistant to echinocandins [12,57]. The mutated residues are conserved in most fungal glucan synthases including *S*. *pombe* Bgs4. This necessitates development of new drug targets for anti-fungal therapeutics. Sbg1 is a small protein that has a domain conserved in all known pathogenic fungi (S1C Fig). Thus, our studies on Sbg1 in fission yeast will help us not only understand cytokinesis but also develop a novel drug target.

## Materials and Methods

### Yeast strains and genetic methods

We used PCR-based gene targeting and standard genetic methods to construct strains [58,59]. All tagged strains are regulated under native promoters and integrated into endogenous chromosomal loci except the strains for Sbg1 depletion that are under the control of thiamine repressible *nmt1* promoters [37,38]. To tag Sbg1 at its N-terminus under the control of the *sbg1* promoter, we replaced the *3nmt1* promoter in plasmids pFA6a-kanMX6-P3nmt1-mEGFP (monomeric enhanced green fluorescent protein), pFA6a-kanMX6-P3nmt1-mECitrine, and pFA6a-kanMX6-P3nmt1-tdTomato with *sbg1* 5′UTR plus 200 bps upstream sequences (−329 to +6) at *Bgl*II and *Pac*I sites. The resulting plasmids pFA6a-kanMX6-Psbg1-mEGFP (JQW776), pFA6a-kanMX6-Psbg1-mECitrine (JQW775), and pFA6a-kanMX6-Psbg1-tdTomato (JQW774) were then used as templates for PCR amplification and gene targeting at *sbg1* chromosomal loci just upstream of the ATG [59]. We tested the functionalities of tagged Sbg1 by growing cells on YE5S and YE5S+ phloxin B plates from 23 to 36°C. The strains expressing mEGFP-Sbg1, mECitrine-Sbg1, and tdTomato-Sbg1 resembled wt under tested conditions. But tdTomato-Sbg1 had slightly different localization. Some tdTomato-Sbg1 was found to be stuck in internal membranes (S5B Fig). Thus, mEGFP-Sbg1 and mECitrine-Sbg1 are fully functional and tdTomato-Sbg1 is partially functional.

To analyze functions of essential genes and to determine protein localization in their absence, we carried out tetrad fluorescence microscopy (see the Microscopy section) or random spore assays. One copy of the essential gene was deleted using pFA6a-kanMX6 from a diploid strain with both copies of the genes for localization analyses tagged with GFP or tdTomato. The resulting heterozygous diploids were sporulated on SPA5S plates at 25°C for 2 d. The tetrads were then dissected on YE5S or YE5S + 1.2 M sorbitol and grown at 25°C for 1–3 d before imaging. Alternatively, sporulated diploids were digested with 0.5% glusulase (Perkin Elmer, NEE154001EA) at 23°C for ~24 h, washed twice with 1 ml YE5S, concentrated by centrifugation, and plated on YE5S agar plates and grew for 12 to 24 h before imaging. Extra spores were kept at 4°C for repeating experiments. Cells from germinated wt spores in the population were used as controls. Because fission yeast diploid cells are not stable for long-term storage, only the genotypes of parent strains are listed in S1 Table.

To mistargeting Sbg1 or Bgs1, strains expressing mEGFP-Sbg1 and GFP-Bgs1 were crossed to strains expressing mitochondrial outer membrane protein Tom20 tagged at its C-terminus with GBP [42,43].

### Cellular methods and pharmacological treatments

To depolymerize actin filaments, we treated the cells with DMSO or 100 μM Lat-A (Sigma- L5163) for 10 min as described [60]. We used CK-666 (ChemDiv; [61]) at a final concentration of 100 μM from a 10 mM stock solution in DMSO to inhibit actin assembly for endocytosis. Cells were treated with DMSO or CK-666 for 10 min. To disrupt protein secretion, we treated cells with a final concentration of 10 μM Brefeldin-A (Sigma-B7651) from a 10 mM stock solution in ethanol for 10 min in the dark [62].

To visualize the primary septum, cells were stained with 10 μg/ml Calcofluor for 10 min in the dark. To visualize vacuolar membrane in yeast cells and cells from germinated spores, cells were treated with 16 μM FM4-64 (Molecular probes, T3166) from a 16 mM stock solution in DMSO for 20–30 min before washing with 1 ml YE5S twice and imaging. To visualize vacuolar lumen, we used 100 μM Cell tracker^TM^ Blue CMAC Dye (ThermoFisher Scientific, C2110) from a 10 mM stock solution in DMSO. Cells grown in YE5S + thiamine were washed into EMM5S minimal media (to reduce the background) and then CMAC was added to cells and stained for 30 min. The cells were pelleted, resuspended with 1 ml fresh EMM5S and incubated with shaking for 10 min before imaging. Cells were imaged on bare slides and coverslips after all the treatments mentioned in this section.

### Microscopy

Cells were woken up from −80°C stocks (except the ones from germinated spores), restreaked, and grown for 1–2 d on YE5S plates at 25°C and then inoculated into YE5S liquid media. Cells were transferred to YE5S + thiamine medium for indicated times to further repress *nmt1* promoters. Cells were grown at exponential phase for ∼48 h at 25°C before microscopy, except where noted. For microscopy, cells were collected by centrifugation at 3,000 rpm, washed once with EMM5S medium to reduce autofluorescence, and once with EMM5S plus 5 μM n-propyl-gallate. Most cells were imaged on EMM5S + 20% gelatin pads with 5 μM n-propyl-gallate as previously described [42,63,64]. For experiments in Fig 3B, S3D and S6F Figs, cells were washed with fresh YE5S medium once, then with YE5S + 5 μM n-propyl-gallate once, and ∼5 μl of concentrated cells were spotted on a 35-mm dish with a glass coverslip bottom (0420041500C; Bioptechs). The cells were then covered with a piece of YE5S agar before imaging. For imaging germinating spores using Tetrad Fluorescence Microscopy [64–66], the YE5S or YE5S + 1.2 M sorbitol agar plate with the tetrads was cut out and placed face down on a 35-mm dish with a glass coverslip bottom. The agar piece was then covered by an 18 x 18 mm coverslip to slow down the drying of agar media. The Tetrad Fluorescence Microscopy that combines tetrad dissection with spinning disk confocal microscopy allows us to observe the localization of various proteins in cells with the deletion of essential genes. It also allows us to determine the deletion phenotypes unambiguously during the first several cell division. Typically 4–5 tetrads were imaged simultaneously overnight with automated stages.

Microscopy was carried out at 23.5–25°C except where noted. For imaging at restrictive temperatures of temperature-sensitive mutants, a preheated climate chamber (stage-top incubator INUB-PPZI2-F1 equipped with a UNIV2-D35 dish holder; Tokai Hit, Shizuoka-ken, Japan) was used and cells were prepared in the 35-mm dish and the restrictive temperatures were maintained during sample preparations and imaging.

Plan-Apo objective lenses of 100×/1.4 numerical aperture (NA) were used for all the imaging. For visualizing cell morphology and septum only (including Calcofluor staining), cells were imaged on a Nikon Eclipse Ti inverted microscope (Nikon, Melville, NY) equipped with a Nikon cooled digital camera DS-Ql1. We performed other fluorescence microscopy on two confocal systems as described before [42,63,67]: an UltraVIEW ERS spinning-disk confocal system (Perkin Elmer, Waltham, MA) with 405, 488-, 514-, and 568-nm lasers and an ORCA-AG camera (Hamamatsu, Bridgewater, NJ) with 2 × 2 binning, or a spinning-disk confocal system (UltraVIEW Vox CSUX1 system; Perkin Elmer) with 488-, 515-, and 561-nm solid-state lasers and a back-thinned, electron-multiplying charge-coupled device camera (C9100-13; Hamamatsu) without binning.

### Data analysis

Microscopy data were analyzed using ImageJ (National Institutes of Health [NIH], Bethesda, MD), UltraVIEW, or Volocity (Perkin Elmer) software. Fluorescence images shown in the figures are maximum intensity projections of image stacks at 0.4 to 0.6-μm spacing except where noted. The *p* values were calculated using two-tailed Student’s *t* test.

### Electron microscopy (EM)

To observe cell wall and septum morphology in wt (JW81) and *81nmt1-mECitrine-sbg1* (JW6581) mutant, cells were grown in YE5S + thiamine medium at exponential phase for ∼48 h at 25°C and fixed using 2.5% glutaraldehyde in phosphate buffer (0.1 M sodium phosphate, pH 7.4 with 0.1 M sucrose) for 1 h. The fixed cells were then rinsed 3× with 0.1 M sucrose in the 0.1 M sodium phosphate buffer (pH 7.4), and the pellet was submitted to the Campus Microscopy and Imaging Facility at The Ohio State University for further preparations. The samples were further fixed with 1% osmium tetroxide and embedded in agarose block. Then the sample was dehydrated in a graded series of alcohol and embedded in Epon8 epoxy resin. Thin sections of 70–90 nm were cut using a Leica EM UC6 ultramicrotome. Sections were stained with uranyl acetate and lead citrate and imaged using a FEI Tecnai G2 Spirit transmission electron microscope at 80 kV[68–70].

EM with higher resolution was also done at the Boulder Electron Microscopy Services at University of Colorado, Boulder as described previously [42,66]. Briefly, cells were grown similarly (in YE5S + thiamine medium at exponential phase at 25°C for ∼48 h) and then collected using Millipore filters and frozen with high pressure freezing with the Wohlwend Compact 02 Freezer. Freeze-substitution was carried out in the presence of 2% osmium tetroxide and 0.1% uranyl acetate in acetone. Cells were embedded in Epon-Araldite epoxy resin and serially sectioned at 70 nm spacing. The samples were then post stained with uranyl acetate and lead citrate before observation on a 713 Philips CM100 transmission electron microscope (FEI, Hillsboro, OR).

### IP and western blotting

We carried out IP assays and Western blotting as previously described [63,71–73]. Briefly, GFP-tagged protein expressed at its endogenous level was pulled down from *S*. *pombe* cell extracts using protein G covalently coupled Dynabeads (100.04D; Invitrogen, Carlsbad, CA) with polyclonal anti-GFP antibodies (NB600-308; Novus Biologicals, Littleton, CO).

The protein samples were separated on SDS–PAGE and Western blotting was performed using the following monoclonal antibodies: anti-GFP antibody (11814460001, 1:1000 dilution; Roche, Mannheim, Germany); anti-Flag antibody (F1804; 1:500 dilution; Sigma-Aldrich); and TAT1 antibody (1:20,000 dilution) that detects tubulin, the loading control [74]. Anti-mouse secondary antibody was used at 1:5000 to 1:10,000 dilution.

## Supporting Information

S1 Fig Sbg1 is a conserved protein involved in glucan synthesis. (A) Schematic of domain organization of Sbg1. (B) Sequence alignment of the SKN1 domain in *S*. *pombe* Sbg1 with β-glucan synthesis associated proteins from other fungi. *Sp*-*S*. *pombe*; *Bm-Bipolaris maydis; Ca-Candida albicans; Cg-Candida galbrata; An-Aspergillus nidulans; Af-Aspergillus fumigatus;* and *Cn-Cryptococcus neoformans*. Identical and similar (D/E, I/L/V, K/R, N/Q, and S/T) residues compared with Sbg1 are in red and blue, respectively. (C) Cladogram of SKN1 domains from different fungal species. A phylogenetic cladogram with branch length (indicated by the numbers) showing the relationship of the Sbg1 SKN1 domain with homologous β-glucan synthesis associated proteins from other fungal species. Fungal pathogens are in bold. NCBI’s BLAST and EMBL-EBI’s ClustalW2 were used to generate the cladogram.
(PDF)

S2 Fig *sbg1*Δ leads to cell lysis and defective ring constriction. (A and B) Cell lysis (A) and defective ring constriction and disassembly (B) in *sbg1*Δ cells. *sbg1*Δ spores were dissected and germinated on YE5S plates for 24 h before imaging. (A) Images from a movie with 10 min intervals showing before and after cell lysis. The scale bars (for this and other supplemental figures except the EM images) represent 5 μm. (C) Protein levels of Sbg1 after depletion using *nmt1* promoters. Cell extracts of *81nmt1-mECitrine-sbg1* (left) and *41nmt1-mECitrine-sbg1* (right) cells grown in YE5S + thiamine for indicated times (0 to 72 h) were used to test Sbg1 levels. Lane C, control with mECitrine-Sbg1 expressed from its native promoter. The arrows mark the expected Sbg1 band. Tubulin was used a loading control. (D) Quantification of cell length in wt and *81nmt1-mECitrine-sbg1* cells grown as in Fig 1C. (E) The primary septum is deficient in Sbg1 depletion cells revealed by Calcofluor staining. Cells were grown in YE5S + thiamine for 60 h before staining. (F) Illustration of the measurements of septum and cell-wall thickness on EM images. The septum was measured at three equal distant points (marked by red circles) along the septa. The cell wall was measured at the very cell tip and two sides, which are halfway across the daughter cell (marked by yellow circles). Scale bar represents 1 μm.
(PDF)

S3 Fig Localization dependencies of Sbg1 and partial colocalization of Sbg1 with glucan synthases Bgs4 and Ags1. (A) Quantification of arrival and ring formation of Sbg1 at the division site using Sad1 as a cell-cycle marker. (B) Micrographs of *mEGFP-sbg1* cells treated with DMSO, CK-666, or Lat-A. (C and D) Sbg1 localization depends on the secretory pathway. (C) Cells were treated with ethanol or BFA. (D) Sbg1 localization in wt and *sec8-1* mutant grown at 36°C for 4 h. (E and F) Micrographs (E) and line scans at the dashed lines on the vertical views (F) showing partial colocalization of Sbg1 with glucan synthases Bgs4 and Ags1. Bgs1 was shown for comparison.
(PDF)

S4 Fig Localization and protein levels of Bgs1, Bgs4, and Ags1 in *sbg1* mutants. (A and B) *sbg1Δ* spores were germinated on YE5S plates for 24 h before imaging. (A) Time course showing no localization of GFP-Bgs1 in *sbg1*Δ cells. (B) Quantification of relative GFP-Bgs1 global levels in *sbg1Δ* compared to wt from the same tetrads. (C) Maximum projection and single plane micrographs of GFP-Bgs1 in wt and *41nmt1-sbg1* cells. (D-F) Maximum projection showing localization of Bgs4 (D) and Ags1 (E) and quantification (F) of their global fluorescence intensity in Sbg1 depletion cells. (C-F) Cells were grown in YE5S + thiamine for 36 h.
(PDF)

S5 Fig **Controls for Sbg1 (A) and Bgs1 (B) mislocalization experiments in Fig 4.** Tom20-GBP recruits GFP but not tdTomato tagged proteins to mitochondria. No signal bleedthrough between 488 and 568-nm channels. Bgs1 and Sbg1 colocalization in the first columns is for comparison.
(PDF)

S6 Fig Sbg1 is required for trafficking of Bgs1 but not components in the secretory pathway to the plasma membrane and Cdc15 plays a role in Bgs1 localization. (A, D, E) Cells were grown in YE5S + thiamine for 36 h. (A) Bgs1 accumulates in vacuoles in Sbg1 depletion cells. FM4-64 and CMAC staining of *GFP-bgs1* and *GFP-bgs1 81nmt1-sbg1* cells. (B and C) *sbg1Δ* and wt spores from *sbg1Δ/sbg1*^*+*^ diploid cells expressing homozygous Rlc1-tdTomato with (B) and without (C) GFP-Bgs1 were germinated on YE5S agar plate for 24 h before imaging. Micrographs in (B) and (C) were acquired under the same imaging conditions and processed the same way. Arrows indicate *sbg1Δ* cells. (D and E) Normal localization of the exocyst subunit Sec8 (D) and v-SNARE Syb1 (E) in Sbg1 depletion cells. (F) Sbg1 localization in *cdc15-140* cells grown at 36°C for 3 h. (G) Quantification of relative GFP-Bgs1 global levels in *cdc15*Δ compared to wt from the same tetrads. (H) FM4-64 staining of wt (right cell) and *cdc15Δ* (left cell) cells from spores germinated on YE5S plate for 24 h.
(PDF)

S1 Table *S*. *pombe* strains used in this study. (PDF)

S1 Movie Cell lysis in a *sbg1*Δ cell. *sbg1*Δ spores from tetrad with a plasma membrane marker GFP-Psy1 (green) and a contractile-ring marker Rlc1-tdTomato (red) were dissected and germinated on YE5S plate and the resulting cells were imaged 24 h after the tetrad dissection. The cell was imaged with a time interval of 10 min for ~200 min. Time is in h: min. Frame rate: 7 frames per sec (fps).
(AVI)

S2 Movie Defects in *sbg1*Δ cells are partially rescued by sorbitol. *sbg1*Δ spores with ring marker Rlc1-tdTomato were dissected and germinated on YE5S + 1.2 M sorbitol, imaged 24 h after tetrad dissection with a time interval of 10 min. Time is in h: min. Frame rate: 7 fps.
(AVI)

S3 Movie *sbg1*Δ cells are defective in contractile-ring constriction. *sbg1*Δ spores with a plasma membrane marker GFP-Psy1 (green) and a ring marker Rlc1-tdTomato (red) were dissected and germinated on YE5S plate and the resulting cells were imaged 24 h after tetrad dissection. The cells were imaged with a time interval of 10 min for >10 h. Time is in h: min. Frame rate: 7 frames per sec (fps).
(AVI)

S4 Movie Sbg1 depends on the secretory pathway for its trafficking. Cells expressing mEGFP-Sbg1 were treated with Brefeldin-A (BFA) or its solvent ethanol for ~10 min and imaged on bare slide with a time interval of 1 min. Sbg1 signal in cytoplasmic puncta and cell tips are dramatically reduced. Time is in h: min. Frame rate: 7 fps.
(AVI)

S5 Movie Sbg1 and Bgs1 co-localize and move together in cytoplasmic vesicles. The strain expressing mEGFP-Sbg1 (green, left) and tdTomato-Bgs1 (red, middle) was grown in YE5S at exponential phase for 36 h before imaging continuously (1.1 frames/sec) at a single focal plane. Time is in min: sec. Frame rate: 7 frames per sec (fps).
(AVI)

S6 Movie Bgs1 localization depends on Sbg1. Wt (top) and *sbg1*Δ (bottom) spores expressing GFP-Bgs1 (green) and Rlc1-tdTomato (red) were germinated on YE5S plates and imaged 24 h after tetrad dissection. Cells were imaged with a time interval of 10 min for ~10 h. Time is in h: min. Frame rate: 7 fps.
(AVI)

S7 Movie Bgs1 localization in *cdc15*Δ cells. Wt (top) and *cdc15*Δ (bottom) spores expressing GFP-Bgs1 (green) and Rlc1-tdTomato (red) were germinated on YE5S plates and imaged 24 h after tetrad dissection. Cells were imaged with a time interval of 10 min for ~11 h. Time is in h: min. Frame rate: 7 fps.
(AVI)

## Abbreviations

DIC: differential interference contrast
EM: electron microscopy
ER: endoplasmic reticulum
fps: frame per second
GBP: GFP binding protein
IP: immunoprecipitation
Lat-A: Latrunculin-A
mEGFP: monomeric enhanced green fluorescent protein
mECitrine: monomeric enhanced Citrine
SPB: spindle pole body
tdTomato: tandem dimer Tomato
wt: wild type
