# Supplementary material for: Sbg1 Is a Novel Regulator for the Localization of the β-Glucan Synthase Bgs1 in Fission Yeast
Source: PLoS One. 2016 Nov 29;11(11):e0167043. doi: 10.1371/journal.pone.0167043 (PMC5127554; doi:10.1371/journal.pone.0167043)
Supplement: S1 Fig — (A) Schematic of domain organization of Sbg1. (B) Sequence alignment of the SKN1 domain in S. pombe Sbg1 with β-glucan synthesis associated proteins from other fungi. Sp-S. pombe; Bm-Bipolaris maydis; Ca-Candida albicans; Cg-Candida galbrata; An-Aspergillus nidulans; Af-Aspergillus fumigatus; and Cn-Cryptococcus neoformans. Identical and similar (D/E, I/L/V, K/R, N/Q, and S/T) residues compared with Sbg1 are in red and blue, respectively. (C) Cladogram of SKN1 domains from different fungal species. A phylogenetic cladogram with branch length (indicated by the numbers) showing the relationship of the Sbg1 SKN1 domain with homologous β-glucan synthesis associated proteins from other fungal species. Fungal pathogens are in bold. NCBI’s BLAST and EMBL-EBI’s ClustalW2 were used to generate the cladogram. (PDF) [file pone.0167043.s001.pdf]

Davidson et al., Supplemental Figure 1

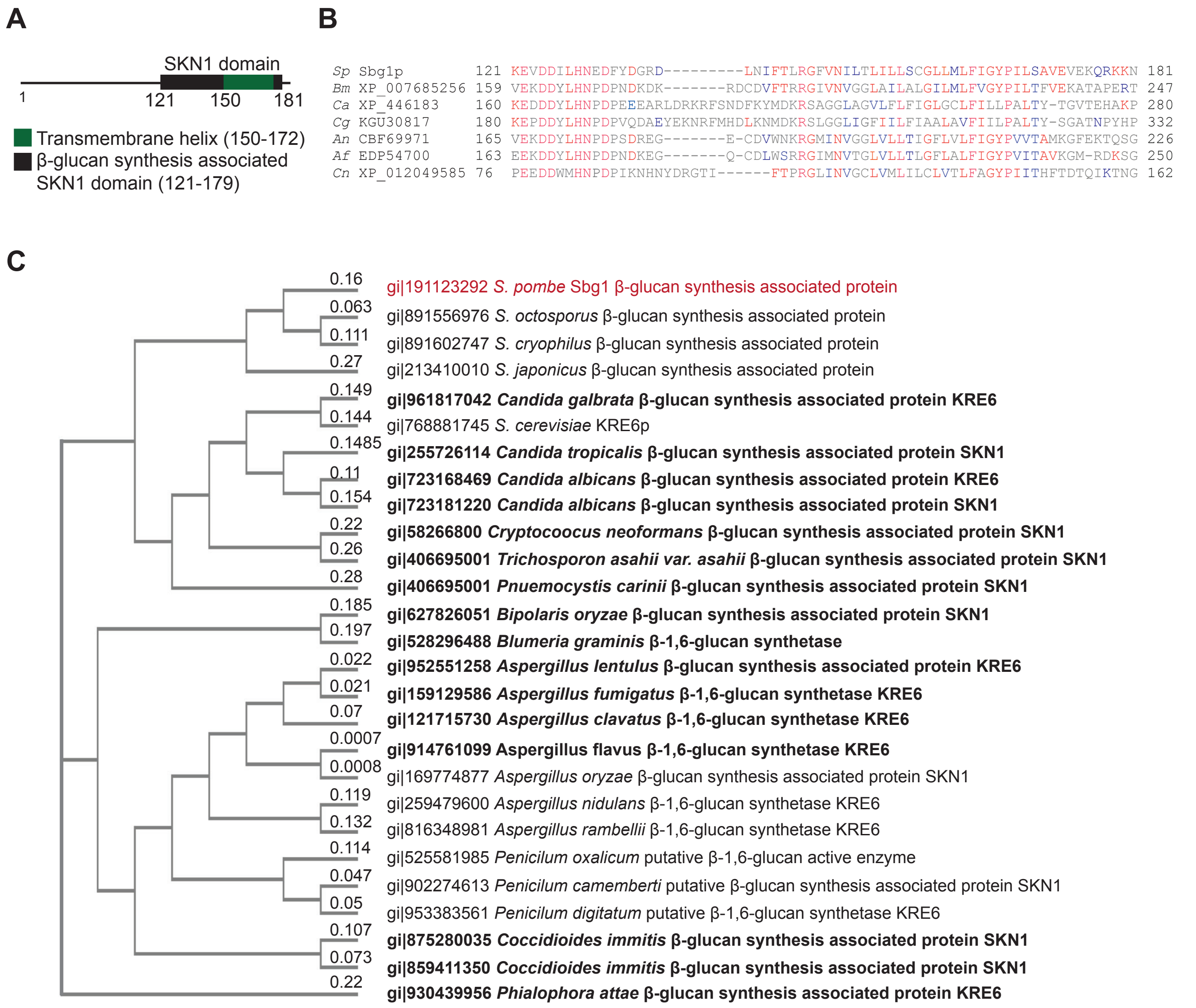

S1 Fig. Sbg1 is a conserved protein involved in glucan synthesis. (A) Schematic of domain organization of Sbg1. (B) Sequence alignment of the SKN1 domain in *S. pombe* Sbg1 with β-glucan synthesis associated proteins from other fungi. *Sp-S. pombe*; *Bm-Bipolaris maydis; Ca-Candida albicans; Cg-Candida galbrata; An-Aspergillus nidulans; Af-Aspergillus fumigatus;* and *Cn-Cryptococcus neoformans*. Identical and similar (D/E, I/L/V, K/R, N/Q, and S/T) residues compared with Sbg1 are in red and blue, respectively. (C) Cladogram of SKN1 domains from different fungal species. A phylogenetic cladogram with branch length (indicated by the numbers) showing the relationship of the Sbg1 SKN1 domain with homologous β-glucan synthesis associated proteins from other fungal species. Fungal pathogens are in bold. NCBI's BLAST and EMBL-EBI's ClustalW2 were used to generate the cladogram.
