# Supplementary material for: Sbg1 Is a Novel Regulator for the Localization of the β-Glucan Synthase Bgs1 in Fission Yeast
Source: PLoS One. 2016 Nov 29;11(11):e0167043. doi: 10.1371/journal.pone.0167043 (PMC5127554; doi:10.1371/journal.pone.0167043)
Supplement: S2 Fig — (A and B) Cell lysis (A) and defective ring constriction and disassembly (B) in sbg1Δ cells. sbg1Δ spores were dissected and germinated on YE5S plates for 24 h before imaging. (A) Images from a movie with 10 min intervals showing before and after cell lysis. The scale bars (for this and other supplemental figures except the EM images) represent 5 μm. (C) Protein levels of Sbg1 after depletion using nmt1 promoters. Cell extracts of 81nmt1-mECitrine-sbg1 (left) and 41nmt1-mECitrine-sbg1 (right) cells grown in YE5S + thiamine for indicated times (0 to 72 h) were used to test Sbg1 levels. Lane C, control with mECitrine-Sbg1 expressed from its native promoter. The arrows mark the expected Sbg1 band. Tubulin was used a loading control. (D) Quantification of cell length in wt and 81nmt1-mECitrine-sbg1 cells grown as in Fig 1C. (E) The primary septum is deficient in Sbg1 depletion cells revealed by Calcofluor staining. Cells were grown in YE5S + thiamine for 60 h before staining. (F) Illustration of the measurements of septum and cell-wall thickness on EM images. The septum was measured at three equal distant points (marked by red circles) along the septa. The cell wall was measured at the very cell tip and two sides, which are halfway across the daughter cell (marked by yellow circles). Scale bar represents 1 μm. (PDF) [file pone.0167043.s002.pdf]

Davidson et al., Supplemental Figure 2

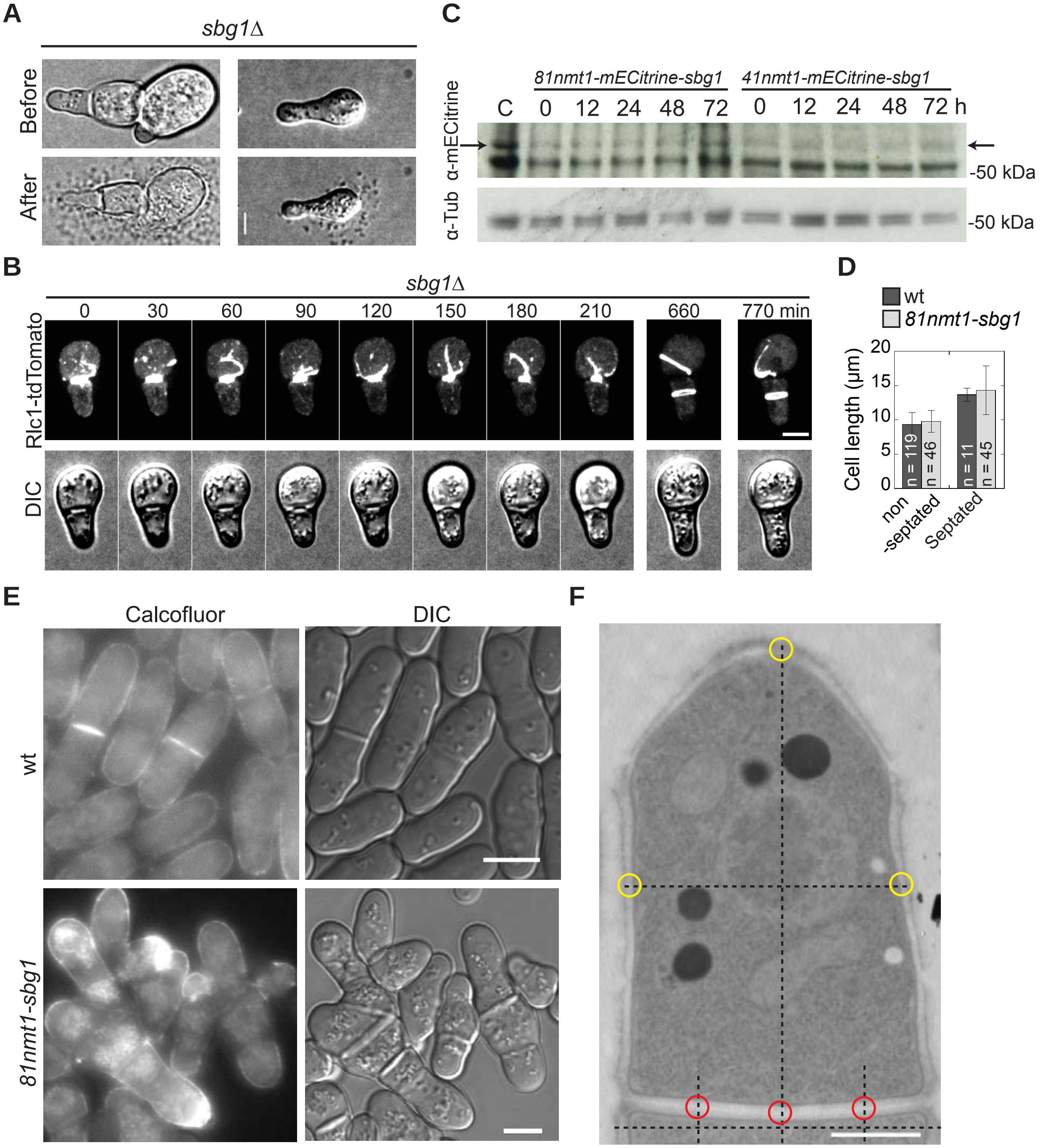

S2 Fig. *sbg1Δ* leads to cell lysis and defective ring constriction. (A and B) Cell lysis (A) and defective ring constriction and disassembly (B) in *sbg1Δ* cells. *sbg1Δ* spores were dissected and germinated on YE5S plates for 24 h before imaging. (A) Images from a movie with 10 min intervals showing before and after cell lysis. The scale bars (for this and other supplemental figures except the EM images) represent 5 µm. (C) Protein levels of Sbg1 after depletion using *nmt1* promoters. Cell extracts of *81nmt1-mECitrine-sbg1* (left) and *41nmt1-mECitrine-sbg1* (right) cells grown in YE5S + thiamine for indicated times (0 to 72 h) were used to test Sbg1 levels. Lane C, control with mECitrine-Sbg1 expressed from its native promoter. The arrows mark the expected Sbg1 band. Tubulin was used a loading control. (D) Quantification of cell length in wt and *81nmt1-mECitrine-sbg1* cells grown as in Fig 1C. (E) The primary septum is deficient in Sbg1 depletion cells revealed by Calcofluor staining. Cells were grown in YE5S + thiamine for 60 h before staining. (F) Illustration of the measurements of septum and cell-wall thickness on EM images. The septum was measured at three equal distant points (marked by red circles) along the septa. The cell wall was measured at the very cell tip and two sides, which are halfway across the daughter cell (marked by yellow circles). Scale bar represents 1 µm.
