# Supplementary material for: Sbg1 Is a Novel Regulator for the Localization of the β-Glucan Synthase Bgs1 in Fission Yeast
Source: PLoS One. 2016 Nov 29;11(11):e0167043. doi: 10.1371/journal.pone.0167043 (PMC5127554; doi:10.1371/journal.pone.0167043)
Supplement: S3 Fig — (A) Quantification of arrival and ring formation of Sbg1 at the division site using Sad1 as a cell-cycle marker. (B) Micrographs of mEGFP-sbg1 cells treated with DMSO, CK-666, or Lat-A. (C and D) Sbg1 localization depends on the secretory pathway. (C) Cells were treated with ethanol or BFA. (D) Sbg1 localization in wt and sec8-1 mutant grown at 36°C for 4 h. (E and F) Micrographs (E) and line scans at the dashed lines on the vertical views (F) showing partial colocalization of Sbg1 with glucan synthases Bgs4 and Ags1. Bgs1 was shown for comparison. (PDF) [file pone.0167043.s003.pdf]

Davidson et al., Supplemental Figure 3

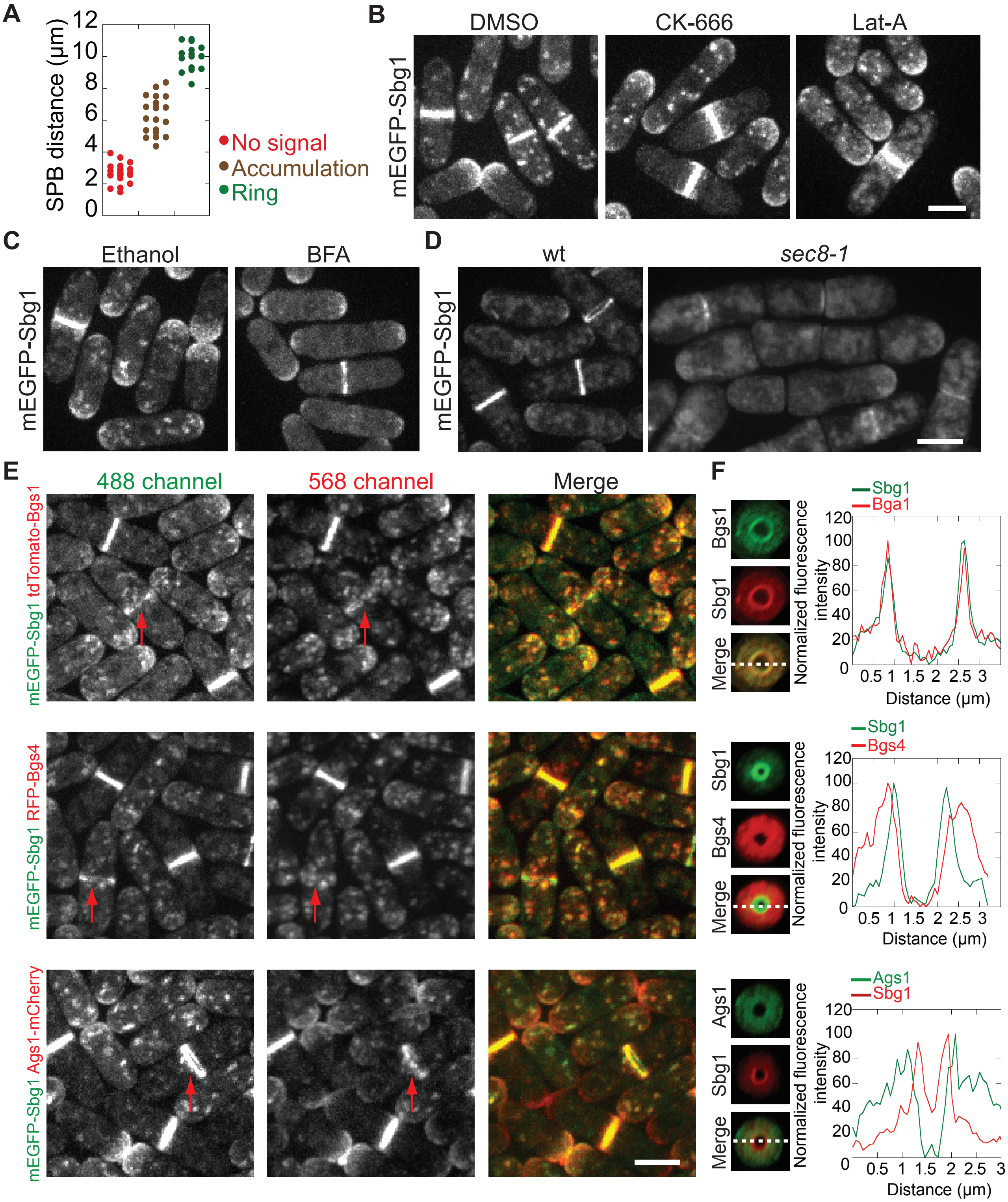

S3 Fig. Localization dependencies of Sbg1 and partial colocalization of Sbg1 with glucan synthases Bgs4 and Ags1. (A) Quantification of arrival and ring formation of Sbg1 at the division site using Sad1 as a cell-cycle marker. (B) Micrographs of *mEGFP-sbg1* cells treated with DMSO, CK-666, or Lat-A. (C and D) Sbg1 localization depends on the secretory pathway. (C) Cells were treated with ethanol or BFA. (D) Sbg1 localization in wt and *sec8-1* mutant grown at 36°C for 4 h. (E and F) Micrographs (E) and line scans at the dashed lines on the vertical views (F) showing partial colocalization of Sbg1 with glucan synthases Bgs4 and Ags1. Bgs1 was shown for comparison.
