# Supplementary material for: Sbg1 Is a Novel Regulator for the Localization of the β-Glucan Synthase Bgs1 in Fission Yeast
Source: PLoS One. 2016 Nov 29;11(11):e0167043. doi: 10.1371/journal.pone.0167043 (PMC5127554; doi:10.1371/journal.pone.0167043)
Supplement: S4 Fig — (A and B) sbg1Δ spores were germinated on YE5S plates for 24 h before imaging. (A) Time course showing no localization of GFP-Bgs1 in sbg1Δ cells. (B) Quantification of relative GFP-Bgs1 global levels in sbg1Δ compared to wt from the same tetrads. (C) Maximum projection and single plane micrographs of GFP-Bgs1 in wt and 41nmt1-sbg1 cells. (D-F) Maximum projection showing localization of Bgs4 (D) and Ags1 (E) and quantification (F) of their global fluorescence intensity in Sbg1 depletion cells. (C-F) Cells were grown in YE5S + thiamine for 36 h. (PDF) [file pone.0167043.s004.pdf]

Davidson et al., Supplemental Figure 4

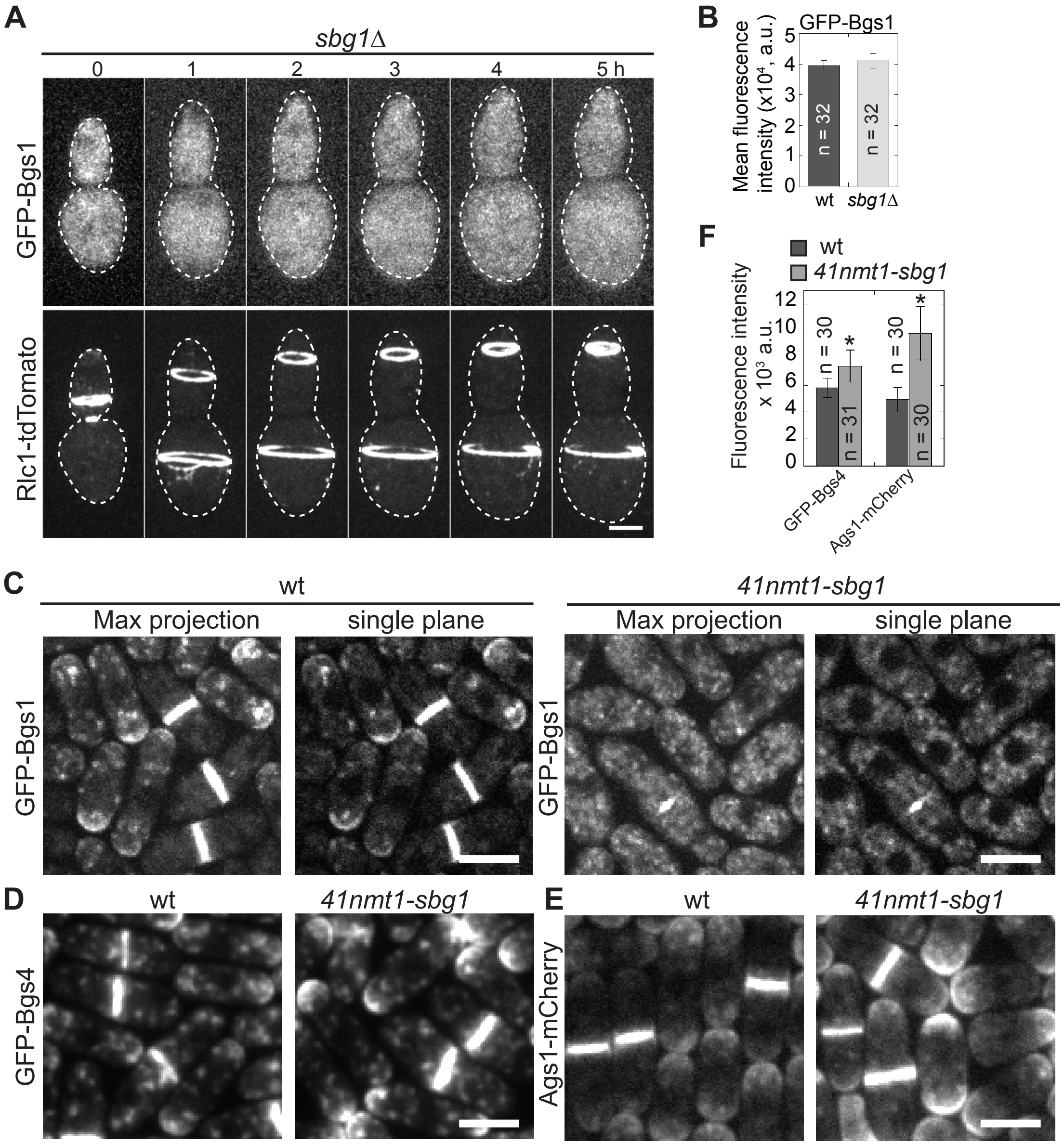

S4 Fig. Localization and protein levels of Bgs1, Bgs4, and Ags1 in *sbg1* mutants. (A and B) *sbg1*Δ spores were germinated on YE5S plates for 24 h before imaging. (A) Time course showing no localization of GFP-Bgs1 in *sbg1*Δ cells. (B) Quantification of relative GFP-Bgs1 global levels in *sbg1*Δ compared to wt from the same tetrads. (C) Maximum projection and single plane micrographs of GFP-Bgs1 in wt and *41nmt1-sbg1* cells. (D-F) Maximum projection showing localization of Bgs4 (D) and Ags1 (E) and quantification (F) of their global fluorescence intensity in Sbg1 depletion cells. (C-F) Cells were grown in YE5S + thiamine for 36 h.
