# Supplementary material for: Sbg1 Is a Novel Regulator for the Localization of the β-Glucan Synthase Bgs1 in Fission Yeast
Source: PLoS One. 2016 Nov 29;11(11):e0167043. doi: 10.1371/journal.pone.0167043 (PMC5127554; doi:10.1371/journal.pone.0167043)
Supplement: S5 Fig — Controls for Sbg1 (A) and Bgs1 (B) mislocalization experiments in Fig 4. Tom20-GBP recruits GFP but not tdTomato tagged proteins to mitochondria. No signal bleedthrough between 488 and 568-nm channels. Bgs1 and Sbg1 colocalization in the first columns is for comparison. (PDF) [file pone.0167043.s005.pdf]

Davidson et al., Supplemental Figure 5

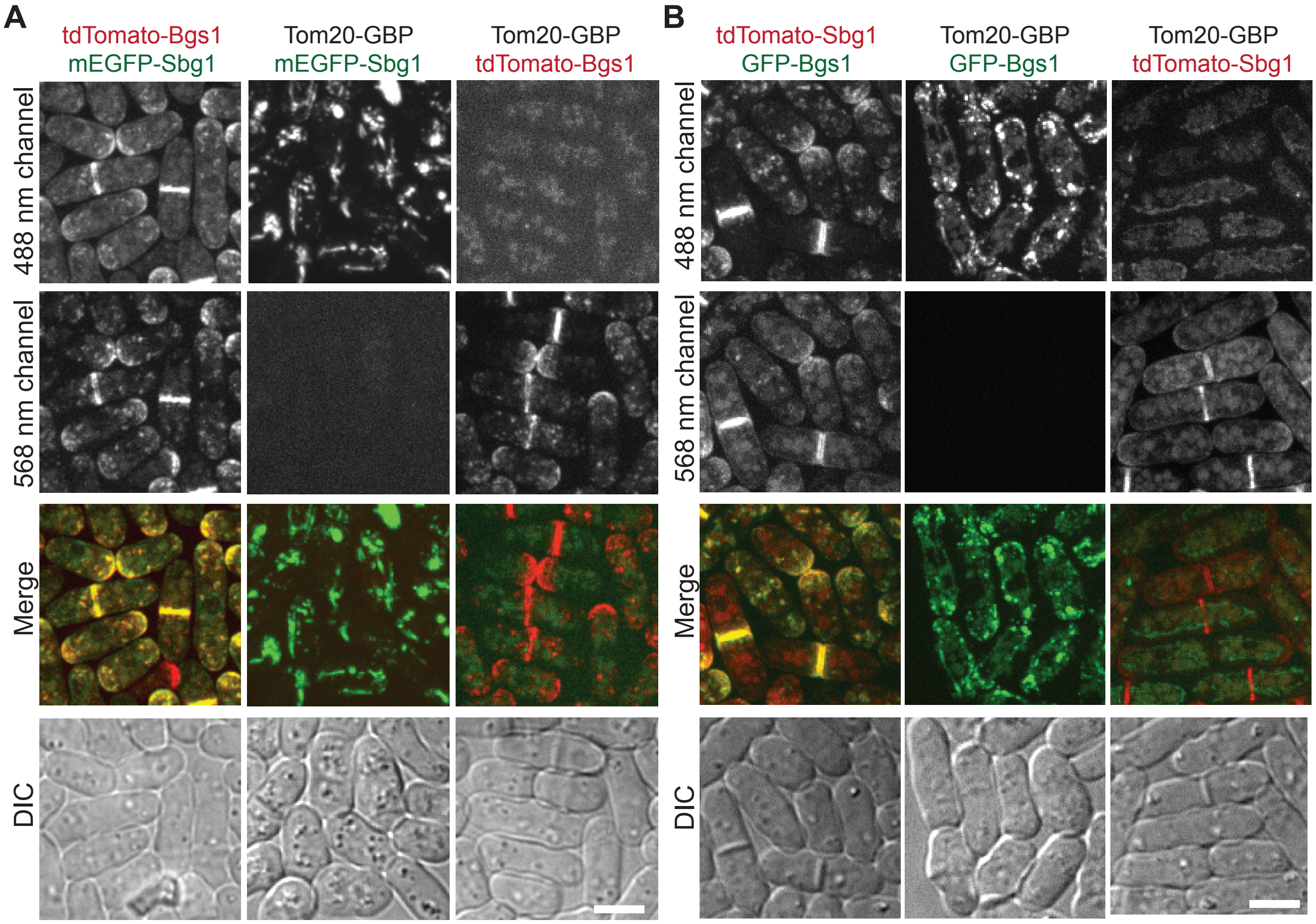

S5 Fig. Controls for Sbg1 (A) and Bgs1 (B) mislocalization experiments in Fig 4. Tom20-GBP recruits GFP but not tdTomato tagged proteins to mitochondria. No signal bleedthrough between 488 and 568-nm channels. Bgs1 and Sbg1 colocalization in the first columns is for comparison.
