# Supplementary material for: Sbg1 Is a Novel Regulator for the Localization of the β-Glucan Synthase Bgs1 in Fission Yeast
Source: PLoS One. 2016 Nov 29;11(11):e0167043. doi: 10.1371/journal.pone.0167043 (PMC5127554; doi:10.1371/journal.pone.0167043)
Supplement: S6 Fig — (A, D, E) Cells were grown in YE5S + thiamine for 36 h. (A) Bgs1 accumulates in vacuoles in Sbg1 depletion cells. FM4-64 and CMAC staining of GFP-bgs1 and GFP-bgs1 81nmt1-sbg1 cells. (B and C) sbg1Δ and wt spores from sbg1Δ/sbg1+ diploid cells expressing homozygous Rlc1-tdTomato with (B) and without (C) GFP-Bgs1 were germinated on YE5S agar plate for 24 h before imaging. Micrographs in (B) and (C) were acquired under the same imaging conditions and processed the same way. Arrows indicate sbg1Δ cells. (D and E) Normal localization of the exocyst subunit Sec8 (D) and v-SNARE Syb1 (E) in Sbg1 depletion cells. (F) Sbg1 localization in cdc15-140 cells grown at 36°C for 3 h. (G) Quantification of relative GFP-Bgs1 global levels in cdc15Δ compared to wt from the same tetrads. (H) FM4-64 staining of wt (right cell) and cdc15Δ (left cell) cells from spores germinated on YE5S plate for 24 h. (PDF) [file pone.0167043.s006.pdf]

Davidson et al., Supplemental Figure 6

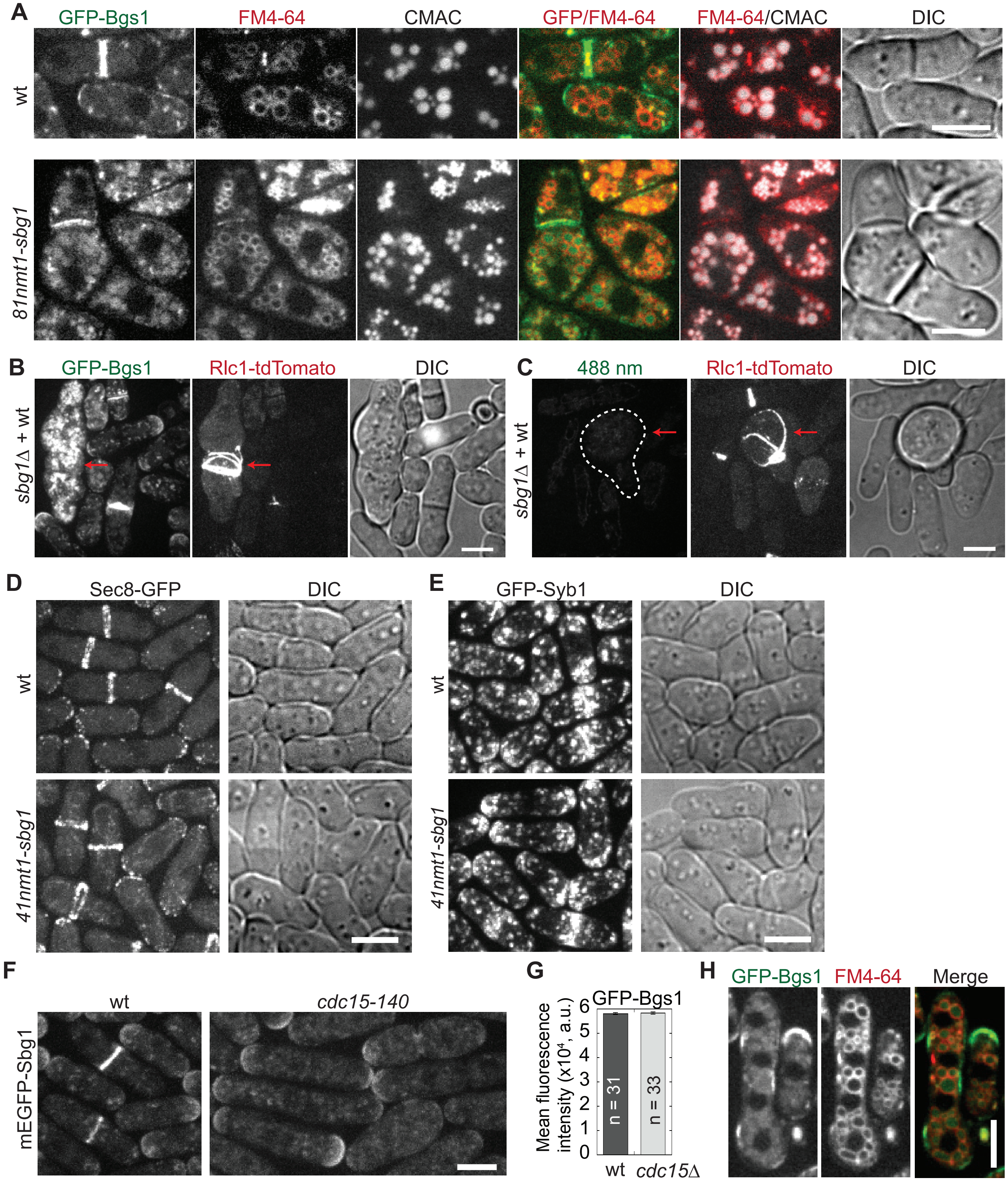

S6 Fig. Sbg1 is required for trafficking of Bgs1 but not components in the secretory pathway to the plasma membrane and Cdc15 plays a role in Bgs1 localization. (A, D, E) Cells were grown in YE5S + thiamine for 36 h. (A) Bgs1 accumulates in vacuoles in Sbg1 depletion cells. FM4-64 and CMAC staining of *GFP-bgs1* and *GFP-bgs1 81nmt1-sbg1* cells. (B and C) *sbg1*Δ and wt spores from *sbg1*Δ/*sbg1*$^{+}$ diploid cells expressing homozygous Rlc1-tdTomato with (B) and without (C) GFP-Bgs1 were germinated on YE5S agar plate for 24 h before imaging. Micrographs in (B) and (C) were acquired under the same imaging conditions and processed the same way. Arrows indicate *sbg1*Δ cells. (D and E) Normal localization of the exocyst subunit Sec8 (D) and v-SNARE Syb1 (E) in Sbg1 depletion cells. (F) Sbg1 localization in *cdc15-140* cells grown at 36°C for 3 h. (G) Quantification of relative GFP-Bgs1 global levels in *cdc15*Δ compared to wt from the same tetrads. (H) FM4-64 staining of wt (right cell) and *cdc15*Δ (left cell) cells from spores germinated on YE5S plate for 24 h.
