# Supplementary material for: Sbg1 Is a Novel Regulator for the Localization of the β-Glucan Synthase Bgs1 in Fission Yeast
Source: PLoS One. 2016 Nov 29;11(11):e0167043. doi: 10.1371/journal.pone.0167043 (PMC5127554; doi:10.1371/journal.pone.0167043)
Supplement: S1 Table — (PDF) [file pone.0167043.s007.pdf]

S1 Table *S. pombe* strains used in this study

Strain	Genotype	Fig/Movie/Reference
JW1341[a]	h^- *rlc1-tdTomato-natMX6 ade6-M210 leu1-32 ura4-D18*	Figs 1A, 1B, 1F, 1G, 1H, S2A, S2B, S6C, and S2 Movie
JW3522[a]	h^+ *rlc1-tdTomato-natMX6 ade6-M216 leu1-32 ura4-D18*	Figs 1A, 1B, 1F, 1G, 1H, S2A, S2B, S6C, and S2 Movie
JW81	h^- *ade6-210 ura4-D18 leu1-32*	Figs 1D, 1E, and S2D
JW6581	h^- *kanMX6-P81nmt1-mECitrine-sbg1 ade6-210 ura4-D18 leu1-32*	Figs 1C, 1D, 1E, S2C, and S2D
JW6169	h^- *kanMX6-P41nmt1-mECitrine-sbg1 ade6-210 leu1-32 ura4-D18*	Fig S2C
JW6687	h^+ *kanMX6-Psbg1-mECitrine-sbg1 ade6-M210 leu1-32 ura4-D18*	Fig S2C
JW6893	*kanMX6-P81nmt1-mECitrine-sbg1 rlc1-tdTomato-natMX6 ade6-M210 leu1-32 ura4-D18*	Figs 1F, 1I, and 1G
JW2042[b]	h^- *rlc1-tdTomato-natMX6 leu1*$^+$*::GFP-psy1 ade6-M210 ura4*	S1 and S3 Movies
JW6872[b]	h^+ *rlc1-tdTomato-natMX6 leu1*$^+$*::GFP-psy1 ade6-M216 leu1-32 ura4-D18*	S1 and S3 Movies
JW81	h^- *ade6-210 ura4-D18 leu1-32*	Figs 2A, 2B, 2D, S2E, and S2F
JW6581	h^- *kanMX6-P81nmt1-mECitrine-sbg1 ade6-210 ura4-D18 leu1-32*	Figs 2A, 2C, 2D, and S2E
JW6687	h^+ *kanMX6-Psbg1-mECitrine-sbg1 ade6-M210 leu1-32 ura4-D18*	Fig 3A
JW6583	*kanMX6-Psbg1-mEGFP-sbg1 sad1-mRFP1-kanMX6 ade6-M210 leu1-32 ura4-D18*	Figs 3B and S3A
JW6787	*kanMX6-Psbg1-mEGFP-sbg1 bgs1Δ::ura4*$^+$ *Pbgs1*$^+$*-tdTomato-bgs1*$^+$*:leu1*$^+$*ade6-M210? his3-D1? leu1-32 ura4-D18*	Figs 3C, 3D, 3E, S3E, S3F, and S5 Movie
JW6174	h^- *kanMX6-Psbg1-mEGFP-sbg1 ade6-210 leu1-32 ura4-D18*	Figs S3B, S3C, S3D, and S4 Movie
JW6661	*sec8-1 kanMX6-Psbg1-mEGFP-sbg1 ade6-210? leu1-32 ura4-D18*	Fig S3D
JW81	h^- *ade6-210 ura4-D18 leu1-32*	Fig 3F
JW6871	h^- *kanMX6-P3nmt1-3FLAG-sbg1 bgs1Δ::ura4*$^+$ *GFP-bgs1-leu1*$^+$ *leu1-32 ura4-D18*	Fig 3F
JW3055	h^- *bgs1Δ::ura4*$^+$ *GFP-bgs1-leu1*$^+$ *leu1-32 ura4-D18*	Fig 3F; [16]
JW6951	*kanMX6-P3nmt1-3FLAG-sbg1 ade6? leu1-32 ura4-D18*	Fig 3F
JW6774	*kanMX6-Psbg1-mEGFP-sbg1 bgs4Δ::ura4*$^+$ *Pbgs4*$^+$*::RFP-bgs4*$^+$*-leu1*$^+$ *ade6-210? leu1-32 his3-D1? ura4-D18*	Figs S3E and S3F
JW6794	*kanMX6-Psbg1-mEGFP-sbg1 ags1Δ 3'UTR*$_{ags1}$$^+$*::ags1*$^+$*-Cherry:leu1*$^+$*:ura*$^+$ *ade6-M210 his3-D1? leu1-32 ura4-D18*	Figs S3E and S3F
JW5249[c]	h^+ *GFP-bgs1-leu1*$^+$ *bgs1Δ::ura4*$^+$ *rlc1-tdTomato-natMX6 ade6-M210 leu1-32 ura4-D18*	Figs 4A, 4B, S4A, S4B, S4C, and S6 Movie
JW6948[c]	h^- *GFP-bgs1-leu1*$^+$ *bgs1Δ::ura4*$^+$ *rlc1-tdTomato-natMX6 ade6-M216 leu1-32 ura4-D18*	Figs 4A, 4B, S4A, S4B, and S6 Movie
JW6174	h^- *kanMX6-Psbg1-mEGFP-sbg1 ade6-210 leu1-32 ura4-D18*	Fig 4D
JW6660	*bgs1-191 kanMX6-Psbg1-mEGFP-sbg1 ade6-210 ura4-D18 leu1-32*	Fig 4D
JW7179	*tom20-GBP-hphMX6 kanMX6-Psbg1-mEGFP-sbg1 bgs1Δ::ura4*$^+$ *Pbgs1*$^+$*-tdTomato-bgs1*$^+$*:leu1*$^+$*ade6-M210? his3-D1? leu1-32 ura4-D18*	Fig 4C

JW7182	*tom20-GBP-hphMX6 kanMX6-Psbg1-tdTomato-sbg1 GFP-bgs1-leu1$^+$ bgs1Δ::ura4$^+$ ade6-M210 leu1-32 ura4-D18*	Fig 4E
JW6892	*GFP-bgs1-leu1$^+$ bgs1Δ::ura4$^+$ rlc1-tdTomato-natMX6 kanMX6-P41nmt1-sbg1 ade6-M210 leu1-32 ura4-D18*	Fig S4C
562	*h$^+$ bgs4Δ::ura4$^+$ Pbgs4$^+$::GFP-bgs4$^+$-leu1$^+$ leu1-32 ura4-D18 his3-D1*	Figs S4D and S4F; [20]
JW7102	*bgs4Δ::ura4$^+$ Pbgs4$^+$::GFP-bgs4$^+$-leu1$^+$ kanMX6-P41nmt1-sbg1 ade6-M210? leu1-32 ura4-D18 his3-D1?*	Figs S4D and S4F
4004	*h$^-$ ags1Δ 3'UTR$_{ags1}$$^+$::ags1$^+$-Cherry:leu1$^+$:ura4$^+$ade6-M210 his3-D1 leu1-32 ura4-D18*	Figs S4E and S4F; [18]
JW7100	*ags1Δ 3'UTR$_{ags1}$$^+$::ags1$^+$-Cherry:leu1$^+$:ura4$^+$ kanMX6-P41nmt1-sbg1 ade6-210 leu1-32 ura4-D18 his3-D1?*	Figs S4E and S4F
JW6787	*kanMX6-Psbg1-mEGFP-sbg1 bgs1Δ::ura4$^+$ Pbgs1$^+$-tdTomato-bgs1$^+$:leu1$^+$ade6-M210? his3-D1? leu1-32 ura4-D18*	Fig S5A
JW7181	*tom20-GBP-hphMX6 kanMX6-Psbg1-mEGFP-sbg1 ade6-M210? his3-D1? leu1-32 ura4-D18*	Fig S5A
JW7180	*tom20-GBP-hphMX6 bgs1Δ::ura4$^+$ Pbgs1$^+$-tdTomato-bgs1$^+$:leu1$^+$ade6-M210? his3-D1? leu1-32 ura4-D18*	Fig S5A
JW7105	*tom20-GBP-hphMX6 GFP-bgs1-leu1$^+$ bgs1Δ::ura4$^+$ade6-M210 leu1-32 ura4-D18*	Fig S5B
JW7531	*kanMX6-Psbg1-tdTomato-sbg1 GFP-bgs1-leu1$^+$ bgs1Δ::ura4$^+$ ade6-M210 leu1-32 ura4-D18*	Fig S5B
JW7532	*tom20-GBP-hphMX6 kanMX6-Psbg1-tdTomato-sbg1 ade6-M210 leu1-32 ura4-D18*	Fig S5B
JW5249[c]	*h$^+$ GFP-bgs1-leu1$^+$ bgs1Δ::ura4$^+$ rlc1-tdTomato-natMX6 ade6-M210 leu1-32 ura4-D18*	Figs 5A, 5B, 5C, 5D, 5E, S6A, and S6H
JW6948[c]	*h$^-$ GFP-bgs1-leu1$^+$ bgs1Δ::ura4$^+$ rlc1-tdTomato-natMX6 ade6-M216 leu1-32 ura4-D18*	Figs 5A and S6H
JW6949	*GFP-bgs1-leu1$^+$ bgs1Δ::ura4$^+$ rlc1-tdTomato-natMX6 kanMX6-P41nmt1-sbg1 ade6-210 leu1-32 ura4-D18*	Fig 5B
JW6891	*GFP-bgs1-leu1$^+$ bgs1Δ::ura4$^+$ rlc1-tdTomato-natMX6 kanMX6-P81nmt1-mECitrine-sbg1 ade6-M210 leu1-32 ura4-D18*	Figs 5C, 5D, 5E, and S6A
JW6088	*sec8-GFP-ura4$^+$ rlc1-tdTomato-natMX6 ade6-M210 leu1-32 ura4-D18*	Fig S6D
JW7104	*sec8-GFP-ura4$^+$ kanMX6-P41nmt1-sbg1 rlc1-tdTomato-natMX6 ade6-M210 leu1-32 ura4-D18*	Fig S6D
JW6151	*h$^-$ GFP-syb1-kanMX6 ade6 leu1 ura$^+$*	Fig S6E
JW7183	*GFP-syb1-kanMX6 kanMX6-P41nmt1-sbg1 ade6 leu1 ura4?*	Fig S6E
JW5249[c]	*h$^+$ GFP-bgs1-leu1$^+$ bgs1Δ::ura4$^+$ rlc1-tdTomato-natMX6 ade6-M210 leu1-32 ura4-D18*	Figs 6A, 6B, 6C, S6B, S6G, S6H, and S7 Movie
JW6948[c]	*h$^-$ GFP-bgs1-leu1$^+$ bgs1Δ::ura4$^+$ rlc1-tdTomato-natMX6 ade6-M216 leu1-32 ura4-D18*	Figs 6A, 6B, 6C, S6B, S6G, S6H, and S7 Movie
JW6174	*h$^-$ kanMX6-Psbg1-mEGFP-sbg1 ade6-210 leu1-32 ura4-D18*	Fig S6F
JW6874	*kanMX6-Psbg1-mEGFP-sbg1 cdc15-140 ade6-M210 leu1-32 ura4-D18*	Fig S6F
1780	*h$^-$ Pbgs1$^+$-tdTomato-bgs1$^+$:leu1$^+$ bgs1Δ::ura4$^+$ leu1-32 ura4-D18 his3-D1*	[17]
FY12587	*h$^-$ leu1$^+$::GFP-psy1 ade6-M210 ura4*	Gift from NBRP, Japan
JW6901	*h$^-$ tom20-GBP-hphMX6 ade6-M210 leu1-32 ura4-D18*	[42]

[a,b,c] The paired strains were used to make different diploids. Diploid strains are not stored long term as they are not stable.
